# Long-term social isolation stress exacerbates sex-specific neurodegeneration markers in a natural model of Alzheimer’s disease

**DOI:** 10.3389/fnagi.2023.1250342

**Published:** 2023-09-20

**Authors:** Carolina A. Oliva, Matías Lira, Claudia Jara, Alejandra Catenaccio, Trinidad A. Mariqueo, Carolina B. Lindsay, Francisco Bozinovic, Grisel Cavieres, Nibaldo C. Inestrosa, Cheril Tapia-Rojas, Daniela S. Rivera

**Affiliations:** ^1^Centro para la Transversalización de Género en I+D+i+e, Vicerrectoría de Investigación y Doctorados, Universidad Autónoma de Chile, Santiago, Chile; ^2^Laboratory of Neurobiology of Aging, Centro de Biología Celular y Biomedicina (CEBICEM), Facultad de Medicina y Ciencia, Universidad San Sebastián, Santiago, Chile; ^3^Centro Científico y Tecnológico de Excelencia Ciencia & Vida, Santiago, Chile; ^4^Centro de Investigaciones Médicas, Laboratorio de Neurofarmacología, Escuela de Medicina, Universidad de Talca, Talca, Chile; ^5^Laboratory of Neurosystems, Department of Neuroscience and Biomedical Neuroscience Institute, Faculty of Medicine, Universidad de Chile, Santiago, Chile; ^6^Center of Applied Ecology and Sustainability (CAPES), Departamento de Ecología, Facultad de Ciencias Biológicas, Pontificia Universidad Católica de Chile, Santiago, Chile; ^7^Departamento de Zoología, Facultad de Ciencias Naturales y Oceanográficas, Universidad de Concepción, Concepción, Chile; ^8^Center of Aging and Regeneration UC (CARE-UC), Departamento de Biología Celular y Molecular, Facultad de Ciencias Biológicas, Pontificia Universidad Católica de Chile, Santiago, Chile; ^9^Centro de Excelencia en Biomedicina de Magallanes (CEBIMA), Universidad de Magallanes, Punta Arenas, Chile; ^10^GEMA Center for Genomics, Ecology and Environment, Facultad de Ciencias, Ingeniería y Tecnología, Universidad Mayor, Santiago, Chile

**Keywords:** *Octodon degus*, social isolation stress, Amyloid-β protein, tau protein, inflammation, oxidative stress

## Abstract

Social interactions have a significant impact on health in humans and animal models. Social isolation initiates a cascade of stress-related physiological disorders and stands as a significant risk factor for a wide spectrum of morbidity and mortality. Indeed, social isolation stress (SIS) is indicative of cognitive decline and risk to neurodegenerative conditions, including Alzheimer’s disease (AD). This study aimed to evaluate the impact of chronic, long-term SIS on the propensity to develop hallmarks of AD in young degus (*Octodon degus*), a long-lived animal model that mimics sporadic AD naturally. We examined inflammatory factors, bioenergetic status, reactive oxygen species (ROS), oxidative stress, antioxidants, abnormal proteins, tau protein, and amyloid-β (Aβ) levels in the hippocampus of female and male degus that were socially isolated from post-natal and post-weaning until adulthood. Additionally, we explored the effect of re-socialization following chronic isolation on these protein profiles. Our results showed that SIS promotes a pro-inflammatory scenario more severe in males, a response that was partially mitigated by a period of re-socialization. In addition, ATP levels, ROS, and markers of oxidative stress are severely affected in female degus, where a period of re-socialization fails to restore them as it does in males. In females, these effects might be linked to antioxidant enzymes like catalase, which experience a decline across all SIS treatments without recovery during re-socialization. Although in males, a previous enzyme in antioxidant pathway diminishes in all treatments, catalase rebounds during re-socialization. Notably, males have less mature neurons after chronic isolation, whereas phosphorylated tau and all detectable forms of Aβ increased in both sexes, persisting even post re-socialization. Collectively, these findings suggest that long-term SIS may render males more susceptible to inflammatory states, while females are predisposed to oxidative states. In both scenarios, the accumulation of tau and Aβ proteins increase the individual susceptibility to early-onset neurodegenerative conditions such as AD.

## 1. Introduction

The lifespan of any organism is finite. The biological aging process is characterized by the slow deterioration of functional processes, the increase in mutation-driven diseases, and a long list of dysfunctional mental and cognitive abilities. The subjective experience of aging could be perceived as a waterfall of symptoms that have no clear beginning, but rather a summation of detrimental physical and cognitive features. Dementia and its most common form, Alzheimer’s disease (AD), is caused by a complex interaction between genetic, lifestyle, environmental, and epigenetic factors (Scheltens et al., 2016; Scheiblich et al., 2020; Wang et al., 2021). Collectively, these factors are recently referred to as allostatic load (Armstrong, 2019). Despite decades of research, the population is still far from being aware of the risk aspects of cognitive deterioration. Furthermore, despite technological advances in diagnosis, most technical probes fail to detect early symptomatic stages.

Across the etiology of AD, only a minority of cases, particularly early-onset forms, appear to be primarily genetic, while interactions between genetic and environmental factors appear to be causative in the remaining forms (Coppedè et al., 2006; Dunn et al., 2019). In this context, a lifestyle of prolonged stress has serious, long-lasting negative effects on brain function and behavior, and may also modify and trigger disease susceptibility (Gubert et al., 2020). There are several types of stress with different biological effects: environmental stress (e.g., toxicants/pollutants, climatic extremes, noise), physical stress (e.g., malnutrition, sleep deprivation, strenuous exercise), and psychological stress (e.g., social isolation, chronic anxiety, fear, excessive cognitive demands) (Karl et al., 2018; Gubert et al., 2020; Wang et al., 2021). The activation of the hypothalamic–pituitary axis (HPA axis) by chronic or uncontrolled exposure to environmental stressors leads to negative physical and mental consequences (Lupien et al., 2009) and also can modulate the pathogenesis of a variety of neurological or neurodegenerative diseases (Gubert et al., 2020). Exposure to stress can trigger and accelerate the cellular, molecular, and behavioral hallmarks of AD (Caruso et al., 2018; Gubert et al., 2020). Consistently, patients affected by AD showed an increase in cortisol levels (Peskind et al., 2001; Curto et al., 2017), while under persistent stress stimuli, they can release pro-inflammatory cytokines in the nervous system, which can lead to neuronal dysfunction and even death (Ricci et al., 2012). Among the common mechanisms proposed between stress and AD are the altered expression and function of amyloid-β, tau hyperphosphorylation and aggregation, as well as neuroinflammation (Carroll et al., 2011; Gubert et al., 2020). Additionally, evidence shows that glucocorticoids and/or corticotropin-releasing hormone (CRH) acting as mediators could contribute to the pathology (Green et al., 2006; Carroll et al., 2011; Futch et al., 2017).

Recently, social isolation and the stress caused by being in isolation emerged as a relevant risk factor for a range of negative mental health outcomes. Social isolation can lead to substantial stress, which in turn can have detrimental effects on both the body and the brain (Shafighi et al., 2023). In response, the brain may release glucocorticoids, leading to a potential impact on neuronal communication and survival (Green et al., 2006; Ouanes and Popp, 2019; Dromard et al., 2022). Additionally, social isolation can induce changes in the structure and function of the brain, resulting in a reduction in the number and complexity of dendrites (Silva-Gómez et al., 2003; Xiong et al., 2023). This, in turn, can impair neuronal communication, leading to detrimental effects on cognitive and emotional functioning (Kline and Mega, 2020) and ultimately contributing to the development of symptoms associated with depression and anxiety, both of which are commonly experienced by socially isolated individuals (Kline and Mega, 2020; Muntsant and Giménez-Llort, 2022).

A promising animal model for the study of the effects of prolonged social isolation is the degu (*Octodon degus*). Previous studies conducted in degus using physiological, behavioral, and electrophysiological approaches, indicated that long-term social isolation stress alters the HPA axis negative feedback loop, affects cognitive performance, the social novelty preference, and increases anxiety like-behavior (Rivera et al., 2020, 2021). Additionally, changes in the expression profile of certain proteins have been observed in a sex-specific manner, indicating both transient and permanent effects depending on the duration of stress exposure (Rivera et al., 2020, 2021). Not only female and male degus show differences in spatial memory acquisition but also experience social stress differently. Early separation of females from littermates had a significant impact on social recognition memory compared to males, indicating that females may be more susceptible to social isolation stress (Rivera et al., 2021). However, at the level of synaptic plasticity, there was no effect of social stress on females, but there was a pronounced effect on males, suggesting that males are more vulnerable (Rivera et al., 2020). Therefore, social isolation stress affected the cognitive performance of the male degus, while the affective and social memory is more affected in the female degus. These findings suggest that prolonged social isolation would raise susceptibility to early-onset neurological and neurodegenerative disease. However, to date, there has been no study that has evaluated whether long-term chronic stress is capable of accelerating the neurodegenerative process in this long-lived animal model. These studies would be of great importance if the interaction with gender could potentially explain why the incidence of dementia is higher in women than in men (Prince et al., 2012; Ardekani et al., 2016; Albert and Newhouse, 2019; Shafighi et al., 2023).

Furthermore, degus are also widely regarded as a natural model of aging, exhibiting several features associated with dementia and AD (Tarragon et al., 2013). Degus display a significantly high number of AD-like characteristics, including a form of amyloid-β that closely resembles the human peptide sequence, differing by only one amino acid from the rodent form (Inestrosa et al., 2005; Hurley et al., 2018). This amyloid-β form generates deposits or plaques, and together with tau phosphorylation, represents the two primary AD-like features found in degus, similar to humans. Also, during the aging process, degus reported the accumulation of ROS, inflammatory molecules, and the production of abnormal proteins (Lindsay et al., 2020), altogether preceded by early angiopathy related to amyloid deposits in vascular vessels as has been previously reported (van Groen et al., 2011), all subtle effects AD-related.

Hence, the purpose of this study is to evaluate whether different degrees of long-term social isolation stress (SIS), experienced from postnatal and post-weaning stages until adulthood, may contribute to the development of early markers associated with AD or related dementia. Furthermore, the study aims to determine if social buffering through re-socialization can potentially mitigate the effects of reactive stress. This research will be conducted on both female and male degus, to explore potential gender differences in vulnerability to these conditions. This paper is part of a compilation of previously published studies on the effects of long-term SIS on various behavioral, physiological, and biochemical aspects using this promising animal model.

## 2. Materials and methods

### 2.1. Social isolation protocol

**Figure 1:**
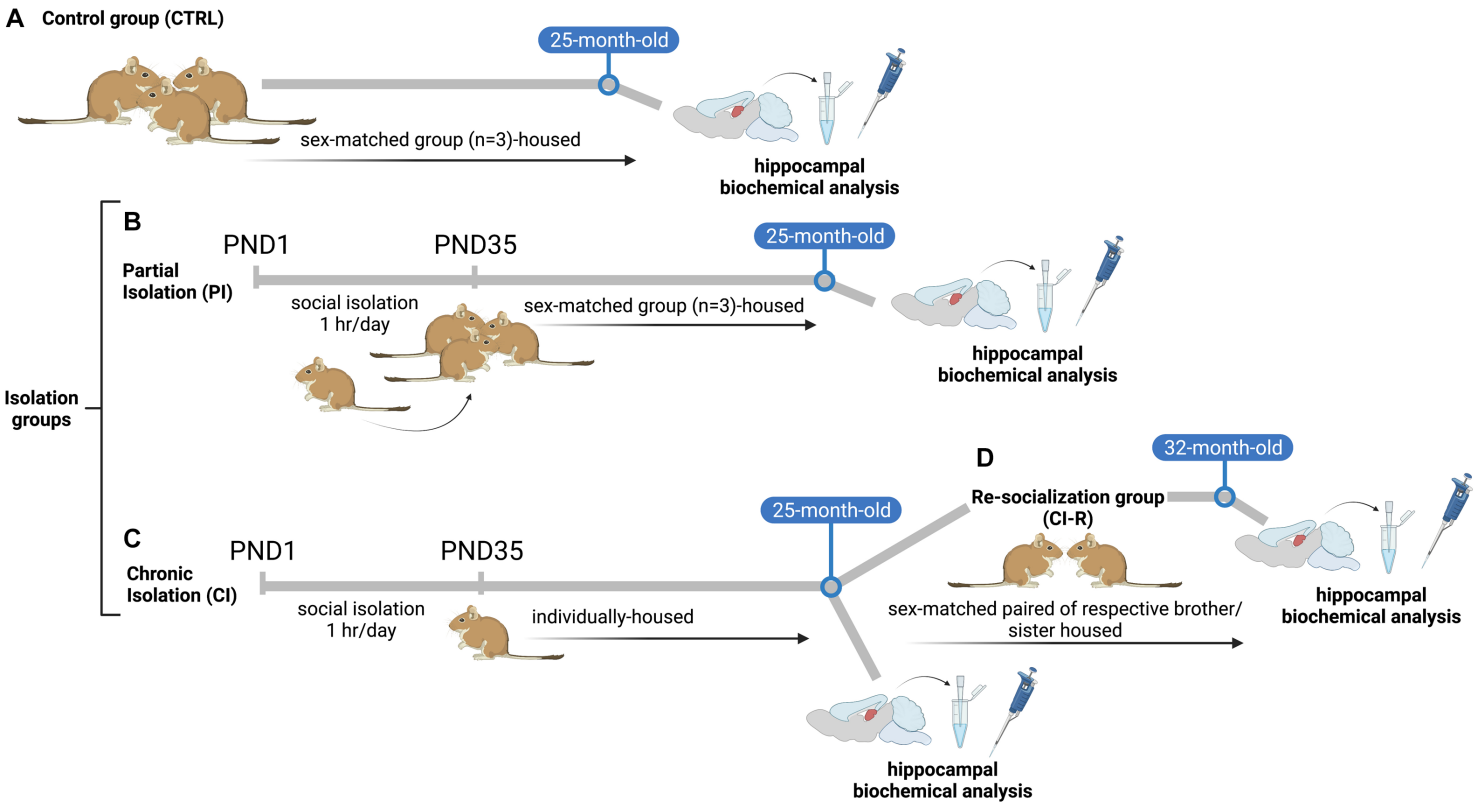
Scheme of experimental design of the stress treatments: **(A)** unstressed control animals (CTRL), where litters were left undisturbed with their family. The siblings remained together until PND 90, and thereafter they were raised in sex-matched groups of three siblings from PND 91 until the end of the experiment **(B)** Partial isolation group (PI), from PND 1 to PND 35 (day of weaning), the degus pups were removed from their mothers and home cage and were kept individually for 1 h daily. After 1-h of separation pups were returned to their family and home cage and left undisturbed until the next day. After PND 35 the whole litter was left undisturbed with their family. The siblings remained together until PND 90, and thereafter they were raised as sex-matched groups of three siblings (one focal degus and two respective brothers/sisters that were not included in our experimental design) from PND 91 until the end of the experiment **(C)** Chronic isolation group (CI), from PND 1 to PND 35 (day of weaning), the degus pups were removed from their mothers and home cage and were kept individually for 1 h daily. After 1-h of separation pups were returned to their family and home cage and left undisturbed until the next day. From PND 36 until the end of the experiment, female and male degus were individually housed **(D)** Re-socialization group (CI-R), after a period of 24-months, CI-reared degus were randomly reassigned and housed in sex-matched pairs with CI-reared brothers or sisters (one focal degus and one sibling not included in our experimental design) during a period of 6-months. Illustration created with BioRender.com.

Pregnant female degus obtained from our colony at the Faculty of Biological Sciences, Pontificia Universidad Catόlica de Chile were kept in pairs and housed in clear acrylic aquaria (length x height x depth: 50 × 35 × 23 cm) with bedding of hardwood chips. Each cage contained one nest box made of clear acrylic (22 × 12 × 15 cm). We checked for litters daily, and the day of birth was defined as PND 0. To avoid litter differential parental effect, the whole litter was randomly assigned to one of the following rearing conditions: (i) unstressed controls: the litters were left undisturbed with their family. The siblings remained together until PND 90, and thereafter they were raised as sex-matched groups of three siblings from PND 91 until the end of the experiment (Control group, CTRL, Figure 1A). (ii) Separation stress: From PND 1 to PND 35 (day of weaning), the pups were removed from their mothers and home cage. In the same room, the pups were kept individually in small opaque cages for 1 h daily (between 09:00 a.m. and 12:00 p.m. noon). Thus, during separation, the pups had acoustic and olfactory but no visual and social contact with their siblings. After 1 h, separated pups were returned to their family and home cage and left undisturbed until the next day. After PND 35 the whole litter was randomly assigned to one of the following rearing conditions: (ii-a) The litters were left undisturbed with their family. The siblings remained together until PND 90, and thereafter they were raised as sex-matched groups of three siblings (one focal degus and two respective brothers/sisters that were not included in our experimental design) from PND 91 until the end of the experiment when degus reached 25-months old (Partial isolation group, PI, Figure 1B). (ii-b) From PND 36 until the end of the experiment, female and male degus were individually housed in standard rodent cages, where they had olfactory, acoustic, and partial visual, but no physical contact to conspecifics (Chronic isolation group, CI, Figure 1C). To study the protective role of social buffering after long-term social isolation stress, after a period of 25-months under CI conditions, both female and male degus were housed in sex-matched pairs with their respective CI-reared brothers or sisters (one focal degus and one sibling not included in our experimental design) during a re-socialization period of 6-month when degus had 31-months old (Re-socialization group, CI-R, Figure 1D).

Animals were kept in a ventilated room exposed to a 12:12 h light–dark cycle with temperatures controlled (yearly minimum = 13.4 ± 0.2°C; yearly maximum = 24.9 ± 0.2°C). Degus were fed a standard rabbit commercial pellet diet (Champion, Santiago, Chile) and *ad libitum* water. The efforts were made to minimize animal suffering and to reduce the number of animals used.

### 2.2. Western blot analysis

Animals were euthanized by decapitation after isoflurane deep anesthesia. The hippocampus was dissected on ice and immediately frozen at −150°C and processed. Briefly, the brain regions were dissected and removed completely by freehand with curved forceps and razorblade (no punch was used) on a petri dish on ice. We separated the hemispheres and the hippocampus was visually identified and removed completely. Tissue samples were homogenized in HEPES buffer (25 mM HEPES, 125 mM NaCl, 1 mM EDTA, 1 mM EGTA, 1% NP-40, pH 7.4) supplemented with a protease inhibitor cocktail (catalog number 78429, Thermo Fisher Scientific) and a phosphatase inhibitor mixture (25 mM NaF, 100 mM Na_3_VO_4_ and 30 μM Na_4_P_2_0_7_) using a glass homogenizer and then passed sequentially through different caliber micropipettes and syringes. Protein samples were centrifuged at 14,000 rpm at 4°C for 20 min and pellets were discarded. Protein concentration was determined using a BCA protein assay kit (Pierce, Thermo Fisher Scientific, Waltham, MA, USA). Samples were resolved by SDS-PAGE, followed by immunoblotting on PVDF membranes. Membranes were incubated overnight with the primary antibody. Then membranes were washed three times with TBS containing 0.1% tween-20 and incubated with HRP-conjugated antibodies (Jackson Immunoresearch, Baltimore, United States). Primary antibodies include mouse anti-catalase (1:1000, sc-271,803, Santa Cruz Biotechnology, Inc., United States), mouse anti-SOD1 (1:1000, sc-271,014, Santa Cruz Biotechnology, Inc., United States), mouse anti-Glutathione reductase (1:1000, sc-133,245, Santa Cruz Biotechnology, Inc., United States), mouse anti-Tau PHF-1 (1:1000 phosphorylated at Ser396 and Ser394) was a gift by Dr. Peter Davies (R.I.P. Department of Pathology, Albert Einstein College of Medicine, NY, United States), mouse anti-pTau clone AT8 (1:1000, MN1020, Invitrogen, United States), mouse anti-human tau (1:1000, 2024-10-31, Dako), rabbit anti-NF-κβ (1:1000, PA5-16758, Invitrogen, United States), mouse anti-Iba1 (1:1000, sc-32,725, Santa Cruz Biotechnology, Inc., United States), mouse anti-β-actin (1:1000, sc-47,778, Santa Cruz Biotechnology, Inc.). The western blots were analyzed using ImageJ. The area of each band measured by densitometry was divided by the loading band (actin) area. The areas from the control samples (five) were averaged and then, every control value was divided by the average control value. The other band areas per treatment were divided by the control, and all these relative data were plotted.

### 2.3. Slot blots

Slot-blot assays were performed as previously described (Tapia-Rojas and Inestrosa, 2018). Briefly, the total protein extracts from the hippocampus were used to perform slot blots. The protein concentration in the hippocampal lysate was determined, and 15 μg of protein was spotted on a 0.45-μm^2^ nitrocellulose membrane (Millipore), followed by blocking with 0.5% PBS-Tween-20 (PBS-T) milk and incubation with the correspondent antibody overnight at 4°C. The slot blots were analyzed using ImageJ. The area of each band was measured by densitometry; the areas of the control samples (five) were averaged and then, each control value was compared to the average control value. The other band areas per treatment were compared to the averaged control, and all these relative data were plotted. Primary antibodies include Mouse Anti-4-Hydroxy-2-Nonenal (4HNE, 1:1000, 298,112 US Biological), rabbit anti-Amyloid-β_42_ (1:1000, ab201061, Abcam), mouse anti-β-Amyloid, 17–24 Antibody, clone 4G8 (1:1000, SIG-39200, Biolegends), rabbit Anti-Amyloid Oligomer Antibody A11 (1:1000, AB9234 Merck Millipore), mouse anti-IL1β (1:1000, sc-12,742, Santa Cruz Biotechnology, Inc.), mouse anti-TNFα (1:1000, sc-12,744, Santa Cruz Biotechnology, Inc.).

### 2.4. Measurement of ATP content

ATP content was measured in hippocampal tissue lysates obtained with a Triton buffer (5 mM Tris, 150 mM NaCl, 1 mM EDTA, 1% (v/v) Triton X-100, pH = 7.4) using a luciferin/luciferase bioluminescence assay kit (ATP determination kit no. A22066, Molecular Probes, Invitrogen) (Torres et al., 2021, 2023). The amount of ATP in each sample was calculated from the standard curves and normalized based on the total protein concentration.

### 2.5. Measurement of ROS content

ROS content was measured using the fluorescent dye CM-H2DCFDA. Briefly, hippocampal samples diluted in Triton Buffer were added to a black 96-well plate in duplicate followed by the addition of 25 μM DCF. Then, the plate was incubated for 5 min and examined in a BioTek Synergy HT (Torres et al., 2021).

### 2.6. Histochemistry

Animals were anesthetized and then perfused through the heart with buffer containing saline solution, followed by fixation with 4% paraformaldehyde in 0.1 M phosphate buffer (PB) for 30 min. Brains were post-fixed overnight at 4°C in 4% PFA (Paraformaldehyde, 1.04005, Merck) in PBS 1x and washed 10 min 3 times in PBS 1x. A sucrose gradient was performed (10, 20% 2 h each at room temperature and 30% overnight at 4°C). The whole brain was submerged in OCT Compound (Ref:4583, Sakura). Coronal sections of 30 μm thickness were collected from a Leica Cryostat, from Anterior to Posterior, in a 24-well plate with PBS 1x and kept at 4°C until use. Representative sections of each animal were mounted in Premium Micro Slide (Ref: PC2-302-16, PR PorLab) in PBS 1x and they were allowed to dry at room temperature.

### 2.7. Cressyl violet staining

The slides were washed briefly in tap water to remove any residual and submerged in Cressyl Solution 5 min (0.3% Cressyl Violet C3886, Sigma, 0.1% Acetic Acid 100,063, Merck). Then they were washed in tap water to remove excess stain and immerse in 3 min in 95% Ethanol and 2 min in 100% Ethanol to clarify the stain. Finally, they were submerged 2 times, 5 min in Xilol to differentiation and mounted in Eukitt Quick-hardening mounting medium (03989, Sigma).

### 2.8. Immunofluorescence

The slides were taken out from −20° and left to dry at room temperature, then the immunofluorescence (IF) was performed. Briefly, they were hydrated in PBS 1x and incubated in Blocking/Permeabilization Solution (Triton X-100 0.5%, BSA 5% in PBS 1x) for 1 h at room temperature. Primary antibodies were diluted in this solution and incubated at 4° overnight. After 3 washes in PBS 1x, 10 min each, secondary antibodies were diluted in the same solution 2 h at room temperature and washed again 10 min 3 times in PBS 1x. The slides were covered in Fluoromount-G (Invitrogen, 00–4,958-02) with Hoechst 33342 (I35103C, Invitrogen). Antibodies used were: mouse Anti-4-Hydroxy-2-Nonenal (4HNE, 298,112 US Biological), mouse 17–24 Antibody, clone 4G8 (SIG-39200, Biolegends), rabbit Arc (156,003, synaptic systems), mouse Tau PHF1 (Ser396 and Ser404, a gift by Dr. Peter Davies (Department of Pathology, Albert Einstein College of Medicine, NY, United States), rabbit NF-κβ p65 (Invitrogen, PA5-16758), anti-rabbit Alexa 594 (Invitrogen, A-21207), and anti-mouse Alexa 488 (Invitrogen, A-21202).

### 2.9. Statistical analysis

All data are presented as the mean ± standard error (SEM). To analyze the effect of stress treatment groups in female and male degus, we used one-way ANOVA and non-parametric analysis of variance (Kruskal–Wallis) when the data did not meet the ANOVA assumptions of normality. Fisher’s LSD *post hoc* comparisons were performed to examine the individual main effect of stress treatments. The assumptions of normally distributed data and homogeneous variances were confirmed using Shapiro–Wilk and Levene’s tests, respectively. Statistical analyses were performed using the Statistica (StatSoft, Tulsa, OK) software package. Differences were considered statistically significant at *p* < 0.05.

## 3. Results

### 3.1. Long-term SIS increases the levels of inflammatory markers, an effect that is partially reverted by re-socialization in female and male degus

**Figure 2:**
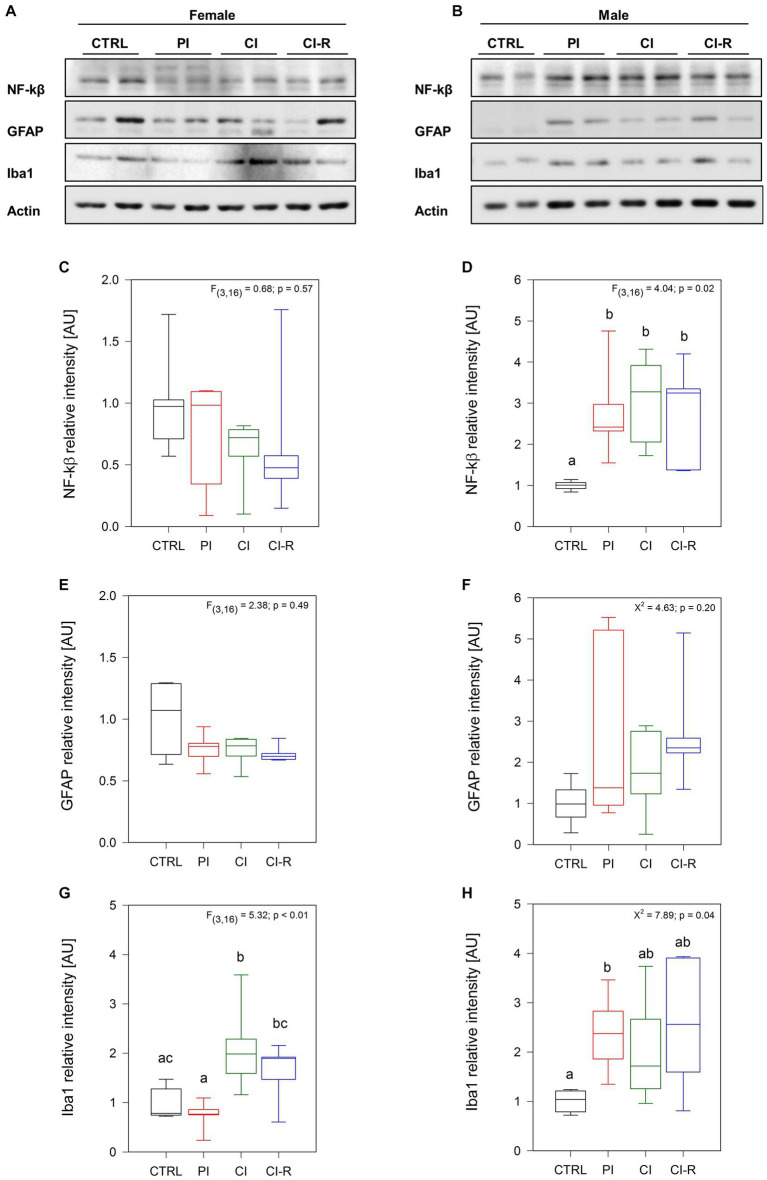
Analysis of inflammatory-related proteins in the hippocampus of female and male degus. Representative western blots of females **(A)** and males **(B)**. Densitometric analyses of NF-kβ protein in female **(C)** and male **(D)** degus, GFAP protein in female **(E)** and male **(F)** degus, and Iba1 protein in female **(G)** and male **(H)** degus. Statistical analysis was performed using one-way ANOVA (*F*-value) or Kruskal–Wallis (*X*^2^), as appropriate. The *p*-value is indicated at the top of the figures. Letters on top of the boxplots indicate significant differences between stress treatments (Fisher’s LSD *post hoc* test). The study included a total of 10 animals (*n* = 5 females, *n* = 5 males).

We measured the nuclear factor kappa β(NF-κβ) a transcription factor involved in a plethora of cellular responses including inflammation, proliferation, and survival (Park and Hong, 2016; Lingappan, 2018). Our data showed no significant effect of long-term SIS treatments on the amount of NF-κβ in females (*p* = 0.57, Figures 2A,C) but a significant effect was observed in males [*F*_(3,16)_ = 4.04, *p* = 0.02; Figures 2B,D]. Specifically, the PI, CI, and CI-R males exhibited higher levels of this inflammatory factor compared to the CTRL group, suggesting that males are more sensitive to stress responses altering NF-κβ levels. In addition, we determined the relative level of the type III-intermediate filament GFAP found in astrocytes, which can indicate abnormal astrocytic remodeling as a consequence of neuronal damage (Shir et al., 2022). Our data showed that neither in females (*p* = 0.49; Figures 2A,E) nor in males (*p* = 0.20; Figures 2B,F) the stress protocols had a significant effect, ruling out astrocytes as the cause of any deleterious effect of long-term SIS. We also measured the levels of ionized-calcium binding adapter molecule 1 (Iba1), a cytoplasmic protein present in microglia and circulating macrophages, which has been correlated with anxiety symptoms and abnormal hippocampal plasticity (Sasaki et al., 2001; Shapiro et al., 2009). Interestingly, we found a significant effect of long-term SIS treatments in females [*F*_(3,16)_ = 5.32, *p* < 0.01; Figures 2A,G] and males [Kruskall–Wallis *X*^2^ = 7.89, *p* = 0.04; Figures 2B,H]. More detailed comparisons indicated that the level of Iba1 is higher in CI females compared to CTRL females, and in PI males compared to CTRL males. During re-socialization, Iba1 levels reach similar values to control in both sexes. Thus, these results suggest that long-term SIS promotes a pro-inflammatory scenario that is more severe in males, but that tends to be attenuated by a period of re-socialization.

**Figure 3:**
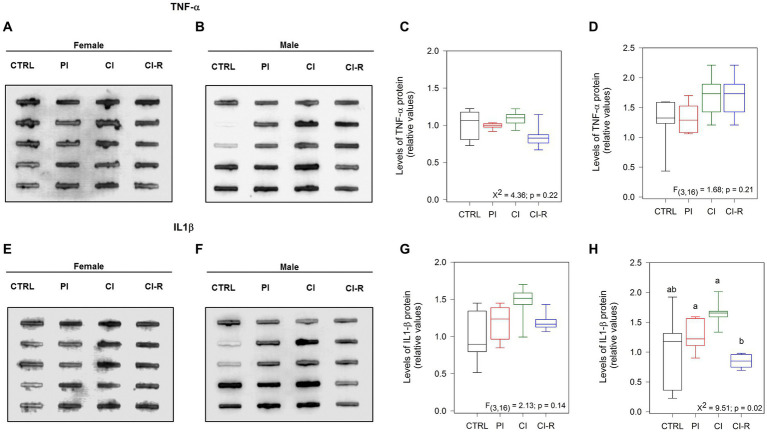
Analysis of cytokines in the hippocampus of female and male degus. Dot blot assay for IL-1β and its corresponding densitometric analysis in the hippocampus of female **(A,C)** and male **(B,D)** degus. Dot blot assay using TNF-α antibody to measure and its corresponding densitometric analysis, in female **(E,G)** and male **(F,H)** degus. Statistical analysis was performed using one-way ANOVA (*F*-value). The *p*-value is indicated at the bottom of the figures. Letters on top of the boxplots indicate significant differences between stress treatments (Fisher’s LSD *post hoc* test). The study included a total of 10 animals (*n* = 5 females, *n* = 5 males).

We also measured the pro-inflammatory cytokines tumor necrosis factor-α (TNF-α) and the interleukin 1β (IL-1β). Both molecules released by microglia, are associated with a pro-inflammatory environment in the brain, and controversial evidences have shown their implications in neurodegeneration and neuroprotection depending on availability, and target cells (Leal et al., 2013). In the case of TNF-α, no differences were observed in females (*p* = 0.22; Figures 3A,C) or males (*p* = 0.21; Figures 3B,D), showing that SIS treatments are not able to trigger an inflammatory response through TNF-α. Regarding IL-1β, we did not find differences in females (*p* = 0.14; Figures 3E,G) but did find it in males (Kruskall–Wallis *X*^2^ = 9.51, *p* = 0.02; Figures 3F,H), being CI-R males significantly lower than the CI males. These data suggest that males could develop more vulnerability to an inflammatory state, and that re-socialization can reduce this susceptibility.

### 3.2. Long-term SIS exerts differential effects on ATP, ROS levels, and oxidative damage in female and male degus

**Figure 4:**
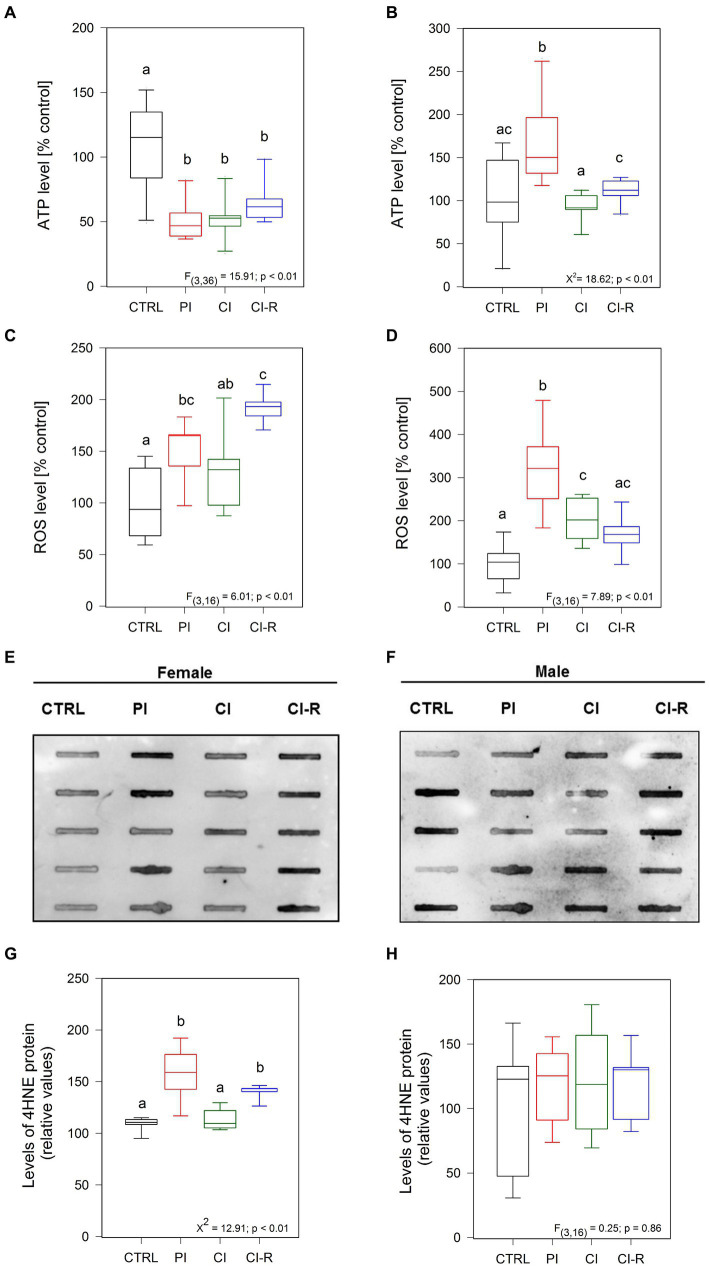
Analysis of ROS and oxidative stress-related proteins in the hippocampus of female and male degus. Panels **(A,B)** show luminescence assay to measure ATP levels in the hippocampus of female **(A)** and male **(B)** degus. Fluorescence assays measuring ROS in female **(C)** and male **(D)** degus, and dot blot analysis of 4HNE protein are presented in panels **(E,G)** for female and **(F,H)** for male degus. Statistical analysis was performed using one-way ANOVA (*F*-value) or Kruskal–Wallis (*X*^2^), as appropriate. The *p*-value is indicated at the bottom of the figures. Letters on top of the boxplots indicate significant differences between stress treatments (Fisher’s LSD *post hoc* test). The study included a total of 10 animals (*n* = 5 females, *n* = 5 males).

Other important factors regulated by isolation stress are the bioenergetics state and the redox balance, both processes mediated mainly by the mitochondria (Möller et al., 2013). For this reason, we quantified ATP using a luciferin/luciferase bioluminescence assay (Torres et al., 2023). The signal obtained is directly proportional to the amount of energy present in the whole lysate of the brain hippocampus of each animal after the long-term SIS protocols. Our data showed significant differences in females [*F*_(3,36)_ = 15.91, *p* < 0.01; Figure 4A] and males (Kruskall–Wallis *X*^2^ = 18.62, *p* < 0.01; Figure 4B). Specifically, all stress-treated groups in females (PI, CI, and CI-R) exhibited significantly lower ATP levels compared to the CTRL group, indicating a detrimental effect of social isolation on cellular energy sources. In contrast, a higher level of ATP was measured in PI males compared to the CTRL group. However, in CI and CI-R males, the ATP levels closely resembled those of the CTRL group. These data suggest that the mechanisms for preserving cellular energy are differentially affected by long-term SIS in females and males.

Next, considering that mitochondria are the main ATP source, we measured the levels of reactive oxygen species (ROS), which are highly unstable oxygen-containing molecules produced during aerobic metabolism (Lennicke and Cochemé, 2021), in the whole hippocampal lysate of all experimental groups (Olesen et al., 2020; Torres et al., 2023). The level of ROS is an indication of the mitochondria status because dysfunctional mitochondria normally increase ROS production. We observed significant differences in ROS among stress treatments in females [*F*_(3,16)_ = 6.01, p < 0.01; Figure 4C] and males [*F*_(3,16)_ = 7.89, *p* < 0.01; Figure 4D]. In particular, significantly high levels of ROS species were measured in the PI groups of both females and males compared to the CTRL groups. Additionally, the ROS level is high in both CI females and males compared to the control ones. Strikingly, while re-socialization led to higher levels of ROS in females, in males, the ROS levels were comparable to the control level. These results suggest that females are more prone to accumulate ROS after long-term SIS, and this is not reversible.

To determine if increased ROS production can induce oxidative damage of lipids, proteins, and DNA molecules, we measured oxidative damage. As a result of lipid peroxidation of polyunsaturated omega-6 acyl groups, 4-hydroxy-2-nonenal (4HNE) accumulates during oxidative stress being linked to the pathology of several diseases, like AD, atherosclerosis, diabetes, and cancer (Breitzig et al., 2016). Using Dot Blot analysis, we quantified the level of 4HNE in the hippocampal lysate. We observed that treatments did affect the level of 4HNE in females (Kruskall–Wallis *X*^2^ = 12.91, *p* < 0.01; Figures 4E,G) but not in males (*p* = 0.86; Figures 4F,H). Specifically, higher level of 4HNE was found in PI females, as well as in CI-R females, indicating that 4HNE stays longer in cells of females after long-term SIS. A similar effect was observed by immunofluorescence, where the 4HNE marker was found higher in the dentate gyrus (DG), CA1, and CA3 from the hippocampus of PI and CI-R female groups (Supplementary Figures 1SA,B). Altogether, these results suggest that females are more sensitive to long-term SIS than males, showing a reduced bioenergetic state and increased oxidative damage.

### 3.3. Differential changes in the protein levels of antioxidant defense after long-term SIS in female and male degus

**Figure 5:**
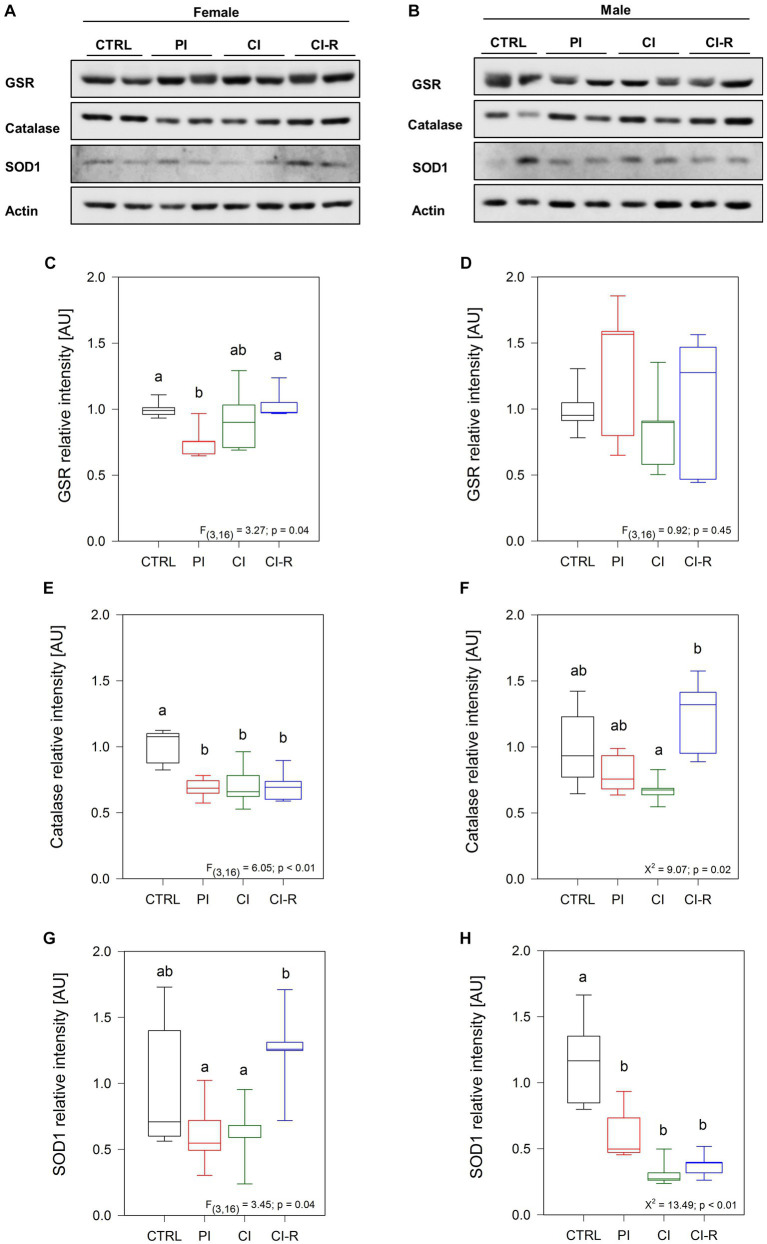
Analysis of antioxidant enzymes in the hippocampus of female and male degus. Representative western blots of females **(A)** and males **(B)**. Densitometric analysis of GSR protein in female **(C)** and male **(D)** degus, catalase protein in female **(E)** and male **(F)** degus, and SOD1 protein of female **(G)** and male **(H)** degus. Statistical analysis was performed using one-way ANOVA (*F*-value) or Kruskal–Wallis (*X*^2^), as appropriate. The *p*-value is indicated at the bottom of the figures. Letters on top of the boxplots indicate significant differences between stress treatments (Fisher’s LSD *post hoc* test). The study included a total of 10 animals (*n* = 5 females, *n* = 5 males).

Increased oxidative damage is caused by increased levels of ROS and reduced antioxidant defenses (Schieber and Chandel, 2014). We evaluated the levels of different antioxidant enzymes in the hippocampus of control and experimental degus (Murphy et al., 2022). First, we measured the levels of glutathione S-reductase (GSR), a central enzyme of cellular antioxidant defense. This enzyme plays a crucial role in reducing oxidized glutathione disulfide (GSSG) to its sulfhydryl form, GSH, which acts as a vital cellular antioxidant (Deponte, 2013). Our data showed significant differences in females [*F*_(3,16)_ = 3.27, *p* = 0.04; Figures 5A,C] but not in males (*p* = 0.45; Figures 5B,D). A significant reduction in GSR levels was observed specifically in PI females, while no significant changes were observed in the other experimental groups. This finding suggests that PI may reduce antioxidant cellular processes. We also measured catalase, an antioxidant enzyme known for efficiently catalyzing the conversion of hydrogen peroxide (H_2_O_2_) into water and oxygen when cells are exposed to environmental stress (Nandi et al., 2019). Our data showed differences in females [*F*_(3,16)_ = 6.05, *p* < 0.01; Figures 5A,E] and in males (Kruskall–Wallis *X*^2^ = 9.07, *p* = 0.02; Figures 5B,F). We observed that all the stress treatments reduced the catalase levels in females. However, in males, the differences were only observed between CI and CI-R males, suggesting that re-socialization can increase catalase levels in males but not in females. In addition, we evaluated the protein levels of superoxide dismutase 1 (SOD1). This cytoplasmic enzyme is relevant for reducing molecular oxygen (O_2_) into peroxide controlling the levels of various ROS and reactive nitrogen species (Schieber and Chandel, 2014). Our data indicates subtle differences in SOD1 levels among females [*F*_(3,16)_ = 3.45, *p* = 0.04; Figures 5A,G] and significant differences among males (Kruskall–Wallis *X*^2^ = 13.49, *p* < 0.01; Figures 5B,H). In detail, CI-R females exhibited higher levels than PI and CI, indicating that re-socialization increases the SOD1 levels. In contrast, all treated male groups showed significantly lower SOD1 compared to the control group. These data suggested that the antioxidant cellular mechanisms during re-socialization in females and males are opposite yet complementary.

### 3.4. Long-term SIS differentially induces the accumulation of abnormal proteins and AD-related tau phosphorylation in female and male degus

**Figure 6:**
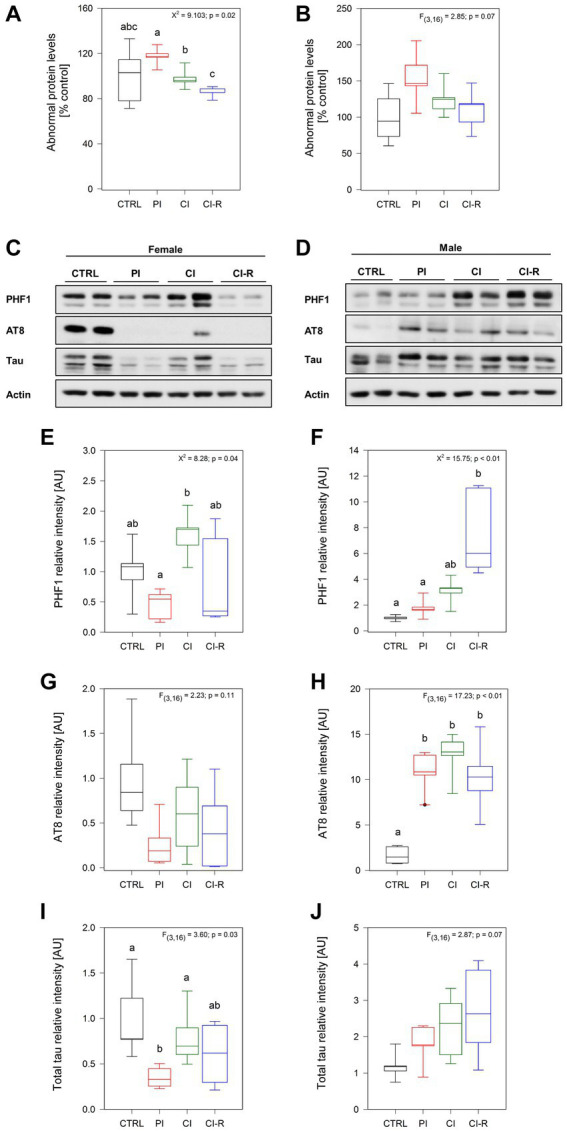
Analysis of abnormal proteins and phosphorylated tau in the hippocampus of female and male degus. Abnormal protein levels in female **(A)** and male **(B)** degus measured by fluorescence in the hippocampus of female and male degus. Representative western blots of phosphorylated and total tau protein in female **(C)** and male **(D)** degus. Densitometric analysis of phosphorylated tau protein in PHF-1 epitope (phosphorylated in Ser396 and Ser404) in female **(E)** and male **(F)** degus, phosphorylated tau at AT8 epitope (Ser202 and Thr205) in female **(G)** and male **(H)** degus, and total tau protein in female **(I)** and male **(J)** degus. Statistical analysis was performed using one-way ANOVA (*F*-value) or Kruskal–Wallis (*X*^2^), as appropriate. The *p*-value is indicated at the top of the figures. Letters on top of the boxplots indicate significant differences between stress treatments (Fisher’s LSD *post hoc* test). The study included a total of 10 animals (*n* = 5 females, *n* = 5 males).

Oxidative damage is the main aging hypothesis and is considered one of the factors inducing the accumulation and aggregation of abnormal proteins in neurodegenerative diseases such as AD (Ross and Poirier, 2004; Spires-Jones et al., 2017). Although not specific, the measurement of abnormal proteins refers to unfolded or misfolded proteins that could be potentially toxic when they aggregate. The cellular inability to eliminate or reverse the misfolding is also indicative of a degenerative disorder (Ross and Poirier, 2004; Spires-Jones et al., 2017). Our data showed significant differences in abnormal proteins in females (Kruskall–Wallis *X*^2^ = 9.103, *p* = 0.02; Figure 6A) but not in males (*p* = 0.07; Figure 6B). In females, each group did not differ from the control group, but significant differences were observed between them, where the PI females showed the highest level and CI-R the lower level. These data suggest that, under re-socialization, females can reduce the level of abnormal proteins caused by chronic stress.

Considering that stress can aggravate the AD phenotype and that degus are a natural model of AD, we evaluated whether this increased load of abnormal proteins will correspond to phosphorylated tau protein or amyloid-β. Tau is a protein associated with microtubules, which act to regulate the microtubule dynamics depending on its phosphorylation state. However, when tau undergoes hyperphosphorylation, this leads to conformational changes and subsequent aggregation (Tapia-Rojas et al., 2019). Here, we evaluated the phosphorylation of tau in two important epitopes present in the brain of degus (Jeganathan et al., 2008). First, we measured the levels of tau phosphorylation at the PHF1 epitope, which recognizes the phosphorylation in Ser396 and Ser404 simultaneously, using a specific antibody (Torres et al., 2021). Our analysis revealed significant differences in females (Kruskall–Wallis *X*^2^ = 8.28, *p* = 0.04: Figures 6C,E) and males (Kruskall–Wallis *X*^2^ = 15.75, *p* < 0.01; Figures 6D,F). More detailed analysis indicated differences between the PI and CI female groups, with the CI groups exhibiting higher phosphorylation levels. In males, the CI-R group showed the most significant increase in phosphorylation compared to the other groups, suggesting a cumulative effect. This effect was also observed by immunofluorescence in Supplementary Figures 1SC,D. Additionally, we quantified the phosphorylation of the tau protein at the AT8 epitope, which recognizes Ser202 and Thr205 phosphorylated residues. Interestingly, we did not observe any significant effect of the treatments in females (*p* = 0.11; Figures 6C,G). However, in males, we found a significant effect [*F*_(3,16)_ = 17.23, *p* < 0.01; Figures 6D,H]. The following analysis showed that in every stress treatment group, males displayed higher levels of AT8 phosphorylated compared to the control ones, suggesting that long-term SIS induced cellular changes that could affect tau folding in males. Finally, we measured the total tau protein, where we observed significant differences in females [*F*_(3,16)_ = 3.6, *p* = 0.03; Figures 6C,I] but not in males (*p* = 0.07; Figures 6D,J). Like with the PHF1, the CI females exhibited a significantly higher level of total tau compared to the PI females. Therefore, these results suggest that long-term SIS induces more severe tau phosphorylation in males compared to female degus. Importantly, this effect persists even during re-socialization.

### 3.5. Long-term SIS promotes increased production and aggregation of Aβ-peptide in both female and male degus

**Figure 7:**
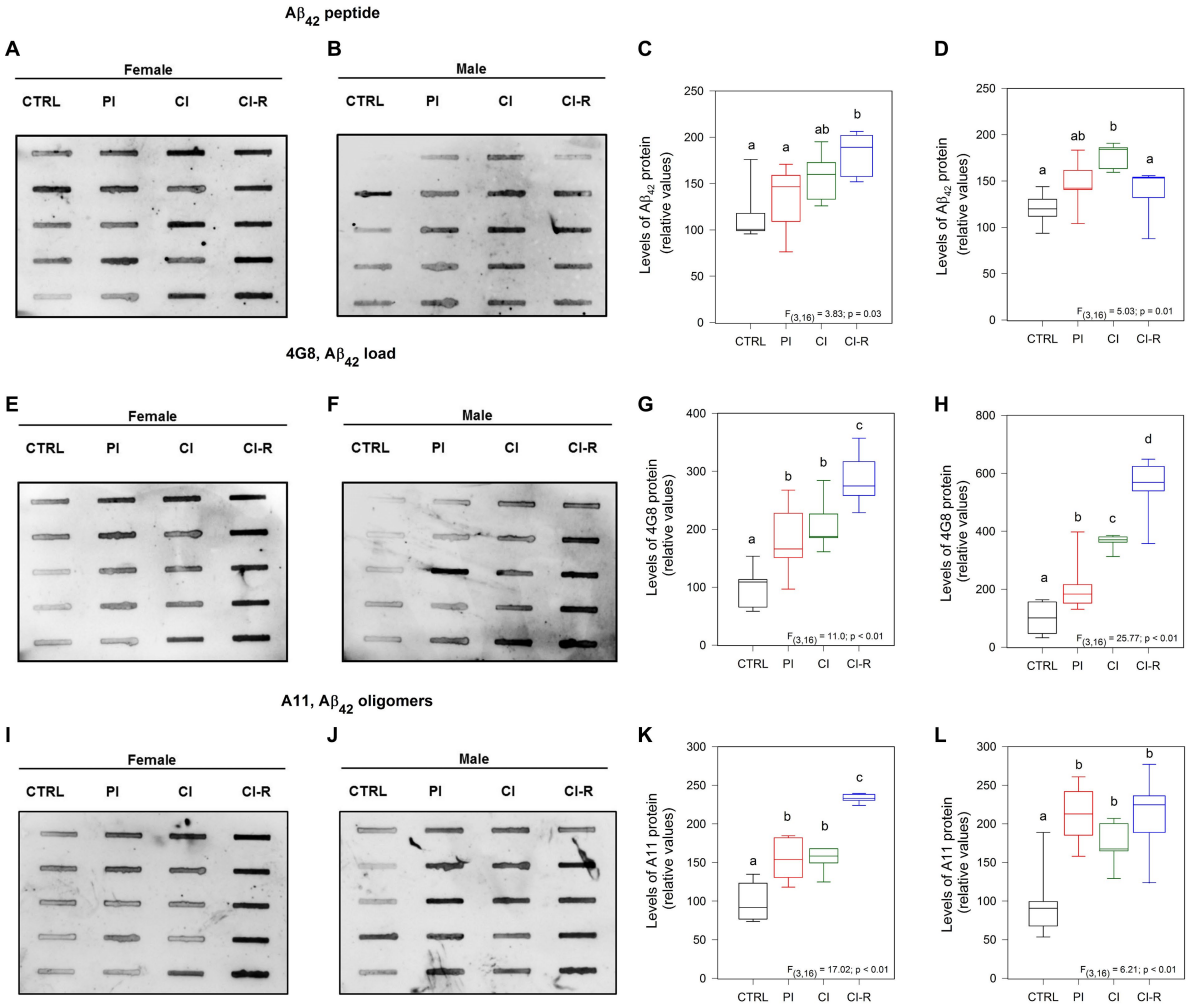
Analysis of amyloid species in the hippocampus of female and male degus. Dot blot assay for Aβ_42_ peptide and its corresponding densitometric analysis, in the hippocampus of female **(A,C)** and male **(B,D)** degus. Dot blot assay using 4G8 antibody to measure Aβ load and its corresponding densitometric analysis, in female **(E,G)** and male **(F,H)** degus. Dot blot assay for Aβ oligomers using the A11 antibody and its corresponding densitometric analysis, in female **(I,K)** and male **(J,L)** degus. Statistical analysis was performed using one-way ANOVA (*F*-value). The *p*-value is indicated at the bottom of the figures. Letters on top of the boxplots indicate significant differences between stress treatments (Fisher’s LSD *post hoc* test). The study included a total of 10 animals (*n* = 5 females, *n* = 5 males).

According to the amyloid hypothesis of AD, Aβ-peptide production and aggregation is the main event responsible for the toxicity in the AD brain hippocampus (Selkoe and Hardy, 2016). For this reason, we measured the levels of Aβ peptide, as well as different aggregation states including Aβ oligomers. We evaluated the presence of this peptide using a specific Aβ_42_ antibody. Our study revealed a significant effect of different long-term SIS treatments in both females [*F*_(3,16)_ = 3.83, *p* = 0.03; Figures 7A,C] and males [*F*_(3,16)_ = 5.03, *p* = 0.01; Figures 7B,D]. Specifically, our data showed that the highest level of Aβ_42_ was observed in CI-R females and in CI males, despite that in males, the Aβ_42_ returned to the control level during re-socialization. These findings suggest that re-socialization may not effectively reduce amyloid accumulation in females.

In addition, we measured the Aβ load using the 4G8 antibody. This antibody specifically recognizes residues 18–23 of the Aβ sequence and has been reported that can recognize amyloid load including aggregates (Hatami et al., 2016). Our data revealed significant effects of treatments on the samples obtained from both females [*F*_(3,16)_ = 11.0, *p* < 0.01; Figures 7E,G] and males [*F*_(3,16)_ = 25.77, *p* < 0.01; Figures 7F,H]. Interestingly, regardless of the treatment, the Aβ load appeared to increase, with higher levels observed in PI, CI, and the highest level in CI-R females compared to the other groups. In males, all the treated groups showed higher levels compared to the CTRL and each other. These findings indicate that long-term SIS leads to an exacerbated release of Aβ levels that cannot be reversed by re-socialization in both sexes, suggesting its accumulation.

We used the A11 antibody, a conformational antibody that specifically recognizes oligomeric forms of Aβ, rather than monomers or fibrils (Chunhui et al., 2018). Our data demonstrated significant effects of long-term SIS treatments in females [*F*_(3,16)_ = 17.02, *p* < 0.01; Figures 7I,K] and males [*F*_(3,16)_ = 6.21, *p* < 0.01; Figures 7J,L]. Further analysis revealed that under all stress treatments, there was an increase in the levels of the oligomeric forms of Aβ in both sexes. Notably, in females, the CI-R group exhibited even higher levels of oligomers compared to the other treatment groups. These findings indicate that long-term SIS treatments induced an exacerbated aggregation of Aβ forming oligomers, and re-socialization was unable to reverse this effect in both sexes.

**Figure 8:**
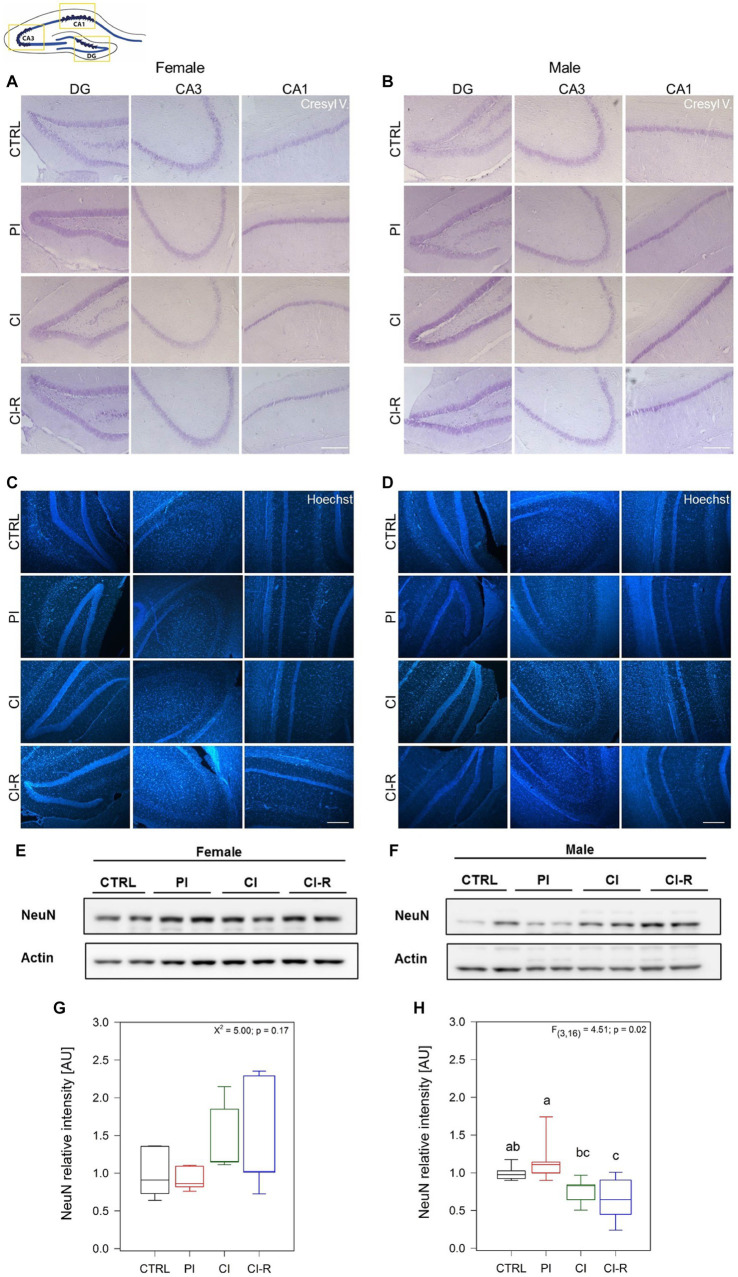
Mature neurons analysis and neural thickness of hippocampal subregions following SIS treatments of female and male degus. Nissl stain (Cressyl violet) in coronal sections of female **(A)** and male **(B)**, and Hoechst stain in female **(C)** and male **(D)** degus. Representative images (63x) obtained using light microscopy, of one animal/sex/condition. Preliminary quantification was performed using Image J software, to measure the neural thickness (μm) of three independent images obtained from the same degus in the DG, CA3, and CA1 hippocampal regions (not shown). Bar scale = 250 μm. The study included a total of 3 samples of hippocampus of one animal for each sex. **(E,F)** Representative western blots of NeuN protein and its corresponding densitometric analysis, in female **(G)** and male **(H)** degus. Statistical analysis was performed using one-way ANOVA (*F*-value). The *p*-value is indicated at the bottom of the figures. Letters on top of the boxplots indicate significant differences between stress treatments (Fisher’s LSD *post hoc* test). The study included a total of 10 animals (*n* = 5 females, *n* = 5 males).

It is known that in advanced stages of the disease, tau hyperphosphorylation and Aβ pathology can promote neuronal death (Yao et al., 2005; Dong et al., 2022). We developed a preliminary study to measure whether SIS treatments could affect neuronal viability. We measured neuronal density using Nissl stain (Figures 8A,B) and Hoechst stain (Figures 8C,D) to load nuclei in the dentate gyrus (DG), CA1, and CA3 regions from the hippocampus of female and male degus. Representative images obtained from one female and male for each experimental group showed a tendency to reduce neural thickness (μm) in the CA1 region of female degus after CI, and in all treated males (Figure 8). To further compare mature cells in our samples, we measured NeuN, a marker of mature neurons, and we found no effect in females (*p* = 0.17) but a significant effect in males [*F*_(3,16)_ = 4.51, *p* = 0.02; Figures 8E–H]. These data suggest a slight vulnerability of males, with lower mature neurons during CI that does not appear to be restored by CI-R.

Thus, in summary, in contrast to the effects observed in neuroinflammatory markers that are reversible, bioenergetic defects, oxidative damage and antioxidant response, as well as the effects on tau phosphorylation and Aβ formation and aggregation appear to be the strongest and most persistent, to accumulate over time, and to affect both sexes, resulting finally in neuronal death. Additional longitudinal studies are needed to elucidate the consequences of long-term SIS and other stressors and their incidence in other neurodegenerative diseases.

## 4. Discussion

*Octodon degus*, a social rodent native to Chile, has emerged as a valuable animal model for studying aging and age-related diseases such as Alzheimer’s disease. One of the most significant advantages of using degus as a model is their relatively long lifespan under captivity conditions, which allows researchers to study the effects of aging and age-related diseases over a longer time frame. Furthermore, degus exhibit cognitive decline and memory impairment like those seen in humans with dementia, and their brains naturally develop amyloid plaques and tau phosphorylation, two hallmarks of Alzheimer’s disease. The molecular and genetic similarities between degus and humans make this animal model ideal for studying the underlying mechanisms of age-related diseases and for testing potential therapeutic interventions. Additionally, by considering both sexes separately, we investigated whether susceptibility to long-term SIS has a sex-dependent component.

### 4.1. Previous reports in female and male degus

**Figure 9:**
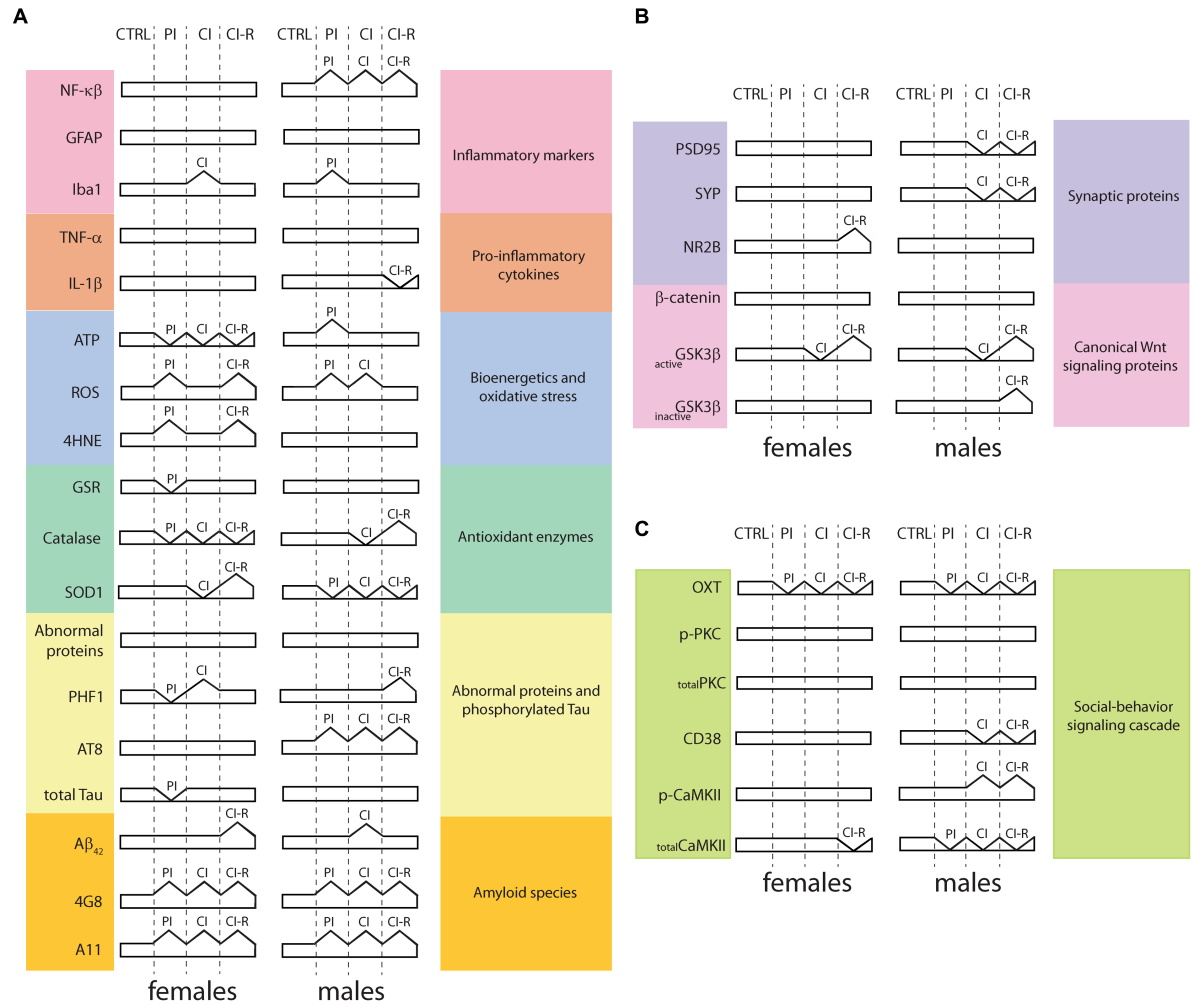
Summary overview of proteins signaling related to long-term SIS treatment in the hippocampus. **(A)** Proteins and enzymes obtained from the present study includes or are included in inflammatory pathways, bioenergetics and oxidative stress, antioxidant enzymes, abnormal proteins, and phosphorylated tau, and amyloid species; **(B)** Data from synaptic proteins and canonical Wnt signaling proteins, obtained in a previous study [modified from Rivera et al., 2020]; **(C)** Data from social-behavior signaling cascade, obtained in a previous study [modified from Rivera et al., 2021]. CTRL: control group, PI: partial isolation group, CI: chronic isolation group, CI-R: re-socialization group, NF-κβ: nuclear factor kappa β, GFAP: type III-intermediate filament, Iba1: ionized-calcium binding adapter molecule 1, TNF-α: tumor necrosis factor-α, IL-1β: interleukin 1β, ATP: adenosine triphosphate, ROS: reactive oxygen species, 4HNE: 4-hydroxy-2-nonenal, GSR: glutathione S-reductase, SOD1: superoxide dismutase 1, PHF1: epitope which recognizes the phosphorylation in Ser396 and Ser404 phosphorylated residues, AT8: epitope which recognizes Ser202 and Thr205 phosphorylated residues, Aβ42: Amyloid β protein fragment 1-42, 4G8: antibody specifically recognizes residues 18-23 of the Aβ sequence, A11: conformational antibody that specifically recognizes oligomeric forms of Aβ, PSD95: postsynaptic density protein 95, SYP: synaptophysin, NR2B: NMDA receptors (NMDAR) containing subunit 2B, GSK3β: Glycogen synthase kinase-3β, activeGSK3β: ratio of pY216-GSK3β/total GSK3β levels, inactiveGSK3β: ratio pS9-GSK3β/total GSK3β levels, OXT: oxytocin, PKC: protein kinase C, p-PKC: PKC phosphorylated at S660, CD38: cluster of differentiation 38, CaMKII: Calcium-calmodulin (CaM)-dependent protein kinase II, p-CaMKII: CaMKII phosphorylated at T286.

We evaluated for the first time in degus the impact of long-term stress induced by social isolation from early life until adulthood on the development and progression of the main neurodegeneration markers. In two previous works, we showed that female and male degus are highly sensitive to long-term SIS treatments (Rivera et al., 2020, 2021). We measured baseline and stress-induced cortisol treatments on the HPA axis (Rivera et al., 2020). We found no differences among animal groups. Because the stress response is expected to be transient, we also measured the “negative feedback” response, which indicates how well an animal decreases cortisol levels after experiencing a stressor. Less negative feedback was found in the CI group compared to the CTRL, while CI-R animals showed significantly higher negative feedback compared to the CTRL ones. These findings suggest that chronic social isolation stress negatively affects the physiological response of the body to normalize the stress response (Rivera et al., 2020) and that social buffering appears to be fundamental for restoring the reduced physiological response to stress (Levy and Yizhar, 2018). Long-term SIS impaired learning performance in both sexes. Using the novel location/object recognition (NOL/NOR) test, we found that working memory was impaired in CI females and males, but recovered during re-socialization. Furthermore, using the Barnes maze, we showed that SIS also elicits a sex-associated stress response on learning and memory, with CI male degus making more reference memory errors (the knowledge of aspects of a task that remain constant between trials) in the stress-treated groups, whereas CI and CI-R females made more working memory errors (the aspects of a task that changes between trials) (Rivera et al., 2020). At the synaptic level, our data showed that synaptic transmission was more efficient in CI males than females but the long-term potentiation (LTP) was unaltered in females despite the long-term SIS treatment compared to males, which was significantly reduced in CI-R (Rivera et al., 2020). A correlation of these effects with hippocampal synaptic proteins was observed, explaining some of these effects, including an increase in NR2B in CI-R females and the reduction of synaptophysisn and PSD95 in CI and CI-R males (Rivera et al., 2020). Later, we investigated the long-term effect of SIS in social-related memory paradigms (Rivera et al., 2021). This study showed that CI males exhibited more anxiety-related behaviors than any of the other treated groups, including females. Meanwhile, the PI females were less motivated to interact with a new animal, as was shown in the social interaction test, suggesting that earlier separation had a stronger effect on females than males, suggesting that this may affect adult behavior. Notably, motivation to engage in novel interactions increased in both female and male CI-R groups. At the molecular level, oxytocin was permanently disrupted in the hippocampus, but also in the hypothalamus and prefrontal cortex in both sexes, showing that the initiation of signaling was impaired in several brain areas. Other molecules such as PKC or CaMKII are increased in the hippocampus of CI and CI-R males, suggesting that males may be able to overcome stressful prior isolation experiences (Rivera et al., 2021). Taken together, the published data demonstrate sex-specific effects of social isolation on social behavior, cognitive memory performance, and molecular outcomes, many of which appear to be irreversible after long-term SIS experience. A concise overview of the protein changes following each long-term SIS treatments, as presented in the current study and the aforementioned references, is shown in Figure 9.

Are there other molecular aspects that could potentially be affected, leading to an increased susceptibility to other diseases? In the present study, we delved into additional metabolic and molecular dimensions to further elucidate this notion.

### 4.2. Inflammatory markers

We analyzed proteins involved in several cellular responses, including inflammation and stress response, among others. Among the factors associated with inflammation, the NF-κβ transcription factor family has been shown to play a role in several pathologies when activated. When present in the nucleus, NF-κβ stimulates the transcription of genes associated with inflammatory processes (Park and Hong, 2016; Lingappan, 2018). Constitutive activation of NF-κβ has been observed in certain types of cancer, suggesting its involvement in promoting cell proliferation and inhibiting apoptosis. Consequently, increased NF-κβ activity may serve as a trigger for abnormal or uncontrolled cellular mechanisms (Park and Hong, 2016). NF-κβ also plays a role in initiating the inflammatory response during cerebral ischemia by activating the release of other cytokines, thus linking its action to cerebrovascular disease and neurodegeneration (Jha et al., 2019). However, it is worth noting that NF-κβ in its inactive constitute form has been detected in active pyramidal neurons in the hippocampus and cerebral cortex, suggesting its involvement in basal synaptic physiology. This indicates that maintaining a balance between anti-inflammatory and pro-inflammatory factors is crucial in determining whether NF-κβ signaling leads to neuroprotection or neurodegeneration (Jha et al., 2019). Overactivation of NF-κβ has been observed in the AD phenotype and its associated symptoms, whereas *in vitro* research has demonstrated the impact of inflammatory factors on increasing the production of Aβ through the NF-κB signaling pathway (Lingappan, 2018).

Our data reveals that under long-term SIS treatments, NF-κβ levels increase in males but not in females, suggesting that social isolation can trigger inflammatory pathways by activating this nuclear transcription factor in a sex-dependent manner. The literature has previously reported sex-dependent gene expression of transcription factors. NF-κβ activity has been reported to be sex-dependent in several tissues. In the brain, the activation of NF-κβ in response to stress-induced neuronal death has a neuroprotective effect bigger in females compared to males (Ruiz-Perera et al., 2018). Recently, a significant increase in the expression of the *NFκβ1* gene and the targets of most NF-κβ family members was found in males with Parkinson’s disease (PD), but not in females (Tranchevent et al., 2023). Indeed, the severity of PD has been associated with NF-κβ activity, which also serves as an indicator of overall mitochondrial function (Laforge et al., 2016). Collectively, these findings suggest that sex-specific dysregulation of certain genes and their downstream targets may play a key role in triggering neurodegeneration.

Sex-associated differences have been reported in mitochondrial processes and cytokine signaling in glial cells (Tranchevent et al., 2023). However, in our experiments, we did not observe any differences in factors such as GFAP between females and males, or by treatment, suggesting that the long-term SIS treatments we applied did not induce astrogliosis. Instead, we observed a significant increase in the expression of the Iba1 protein, which is specific to microglia, in PI males and CI females. The activation of microglia in the brain can be a response to neuronal loss, indicating that males may be more susceptible than females to lower levels of stress. Furthermore, we also observed differences in the IL-1β of males, where re-socialization reduces its levels compared to CI males. This suggests that any inflammatory response caused by early-life chronic isolation can be reversed through re-socialization. Given that blocking or inhibiting this major cytokine could halt the main manifestations of physiological inflammation (Dinarello et al., 2012), re-socialization may represent a unique benefit, at least at early ages. Interestingly, basal circulating IL-1β and TNF-α are higher in men compared to women and their response is also higher in males in several diseases like atherosclerosis and vasculitis (Bernardi et al., 2020). Thus, chronic isolation early in life could increase vulnerability later on in life.

### 4.3. Bioenergetic state, ROS, and oxidative stress

To study the bioenergetic cellular status after long-term SIS treatments, we measured ATP, which measures the amount of energy present in the sample and serves as an indirect measurement of mitochondrial function. Interestingly, we consistently observed lower levels of ATP in all long-term SIS-treated females compared to the control group. This suggests a detrimental effect of social isolation on cellular energy sources in females. In contrast, we found high ATP in PI males compared to the control group, indicating that females and males may employ different mechanisms to preserve cellular energy or that they are differentially affected by long-term SIS. Further studies are required to fully understand and decipher this issue.

To measure oxidative stress, we quantified ROS, which are highly unstable molecules containing oxygen. When ROS combines with other molecules, it can have detrimental effects on DNA and cell survival (Schieber and Chandel, 2014; Lennicke and Cochemé, 2021). Depending on their concentration within cells, ROS can also act as second messengers. However, when ROS levels exceed the cellular mechanisms for clearance, oxidative stress occurs, accelerating aging, and impacting several cellular processes (Dröge and Schipper, 2007; Kregel and Zhang, 2007; Olesen et al., 2020; Torres et al., 2023). In a striking finding, we observed elevated levels of ROS in the PI groups of both sexes. In CI-R groups, ROS levels remained elevated in females but returned to normal in males. This suggests that females may be more sensitive to oxidative stress or have a higher accumulation of ROS. The release of ROS can activate NF-κβ, which in turn up-regulates APP/BACE1, leading to the accumulation of Aβ. At the same time, Aβ secretion stimulates the expression of pro-inflammatory molecules through NF-κβ activation, creating a reverberating cycle of inflammatory processes (Bronzuoli et al., 2016), which is a possible explanation for the high ROS levels in CI-R female degus.

Other molecules, such as the peroxisome proliferator-activated receptor (PPAR)-α, have been implicated in pro-inflammatory processes through NF-κβ signaling. Notably, this transcription factor and ligand-activated nuclear receptor are epigenetically regulated in socially isolated male mice (Matrisciano and Pinna, 2021). The *Ppar-α* gene undergoes hypermethylation, resulting in decreased expression and increased pro-inflammatory factors. This alteration appears to be associated with the aggressive stress-induced behavior observed in isolated mice (Matrisciano and Pinna, 2021). Further data to determine the expression level of this protein or gene in the context of long-term SIS treatments would contribute to a more complete understanding of the physiological response within the SIS paradigm.

As ROS accumulates, other molecules such as 4-HNE, a product of lipid peroxidation, can also accumulate during oxidative stress (Breitzig et al., 2016). In our study, we did not observe differences in the level of 4-HNE in males. However, we found higher level of 4-HNE in the PI and CI-R females. We also observed this effect by immunofluorescence. The presence of this molecule in the PI group suggests that this treatment is particularly sensitive for females, resulting in a higher accumulation of oxidative stress molecules. 4-HNE is highly reactive and forms covalent adducts with other proteins, thereby deactivating key proteins (Shoeb et al., 2013; Breitzig et al., 2016). For example, in mitochondria, it can impair ATPase activity or modify proton transport. Many of its targets are redox-sensitive molecules and it can also upregulate the expression of NF-κβ, thereby affecting processes like inflammation and apoptosis (Breitzig et al., 2016). Furthermore, 4-HNE has been linked to the pathology of several diseases, including AD, atherosclerosis, diabetes, and cancer (Shoeb et al., 2013).

### 4.4. Antioxidants

Resisting oxidative stress is crucial for cells to maintain dangerous reactive species away from physiological processes. The GSR enzyme plays a central role in cellular antioxidant defense by maintaining the reducing environment within the cell (Deponte, 2013; Dwivedi et al., 2020). In our study, we observed a significant reduction in GSR levels in the PI females, while no changes were observed in the other groups of females. Conversely, there were no changes in GSR levels in males. This data suggests that females may be at higher risk of experiencing oxidative stress following partial social isolation. The evidence of sex-dependent oxidative stress is not new, with studies in other species indicating a correlation with longevity (Martínez de Toda et al., 2023). In *Drosophila*, females lived longer, something that is conferred by their resistance to oxidative stress (Niveditha et al., 2017). Similarly, in female rats, the level of GSR is higher compared to males and is influenced by estrogen, similar to findings in humans (Bellanti et al., 2013). It is important to note that our data do not compare females with males but instead examine the effects related to different degrees of stress, showing that females can reestablish GSR levels during chronic isolation.

Another enzyme that showed significant changes in females is catalase, which catalyzes the breakdown of hydrogen peroxide into oxygen and water in all cellular regions where H_2_O_2_ is produced (Nandi et al., 2019). Despite that catalase appears to be similar in most human tissues, including the brain (Martínez de Toda et al., 2023), our samples exhibited a significant reduction in all treatment groups of females compared to the control. In males, we observed differences only between the re-socialized group and the chronically isolated group, indicating that re-socialization may enhance the enzyme and potentially its activity in males but not in females. We also measured the SOD1, an enzyme located in the cytoplasm that catalyzes the conversion of superoxide into oxygen and H_2_O_2_ and that is one step before catalase in the antioxidant defense pathway. Similar to catalase in males, SOD1 levels increased in the resocialized female group compared to the chronically isolated females. Conversely, in males, the level of SOD1 decreases in all treated groups, similar to catalase in female-treated groups. These results suggest that long-term SIS treatments have a greater impact on altering SOD1 levels in males than in females. However, males seem to exhibit a better response during re-socialization, as they increase catalase levels to counteract the presence of peroxide. On the other hand, females respond during re-socialization by increasing the amount of SOD1, but the subsequent step involving catalase does not show a similar reaction. SOD enzymes play a crucial role in limiting the potential toxicity of ROS and nitrogen species released during metabolic activities (Schieber and Chandel, 2014). Additionally, the specificity of SOD enzymes for subcellular compartments indicates their functional relevance.

### 4.5. Abnormal proteins and tau phosphorylation

The measurement of misfolded proteins is not specific to a particular protein but generally refers to the presence of proteins that have adopted abnormal conformations and can potentially form toxic aggregates. The inability of cells to effectively eliminate or reverse protein misfolding is considered indicative of degenerative disorders (Aigelsreiter et al., 2007). In our study, each experimental group reported different levels with the highest abnormal protein level found in PI females and the lowest in the re-socialized female group. These differences may reflect the unique effects of each treatment on protein misfolding and aggregation, highlighting the specific impact of each condition on cellular processes.

Tau is a microtubule-associated protein predominantly expressed in neurons. Abnormal hyperphosphorylation of tau leads to its detachment from microtubules, leading to changes in its spatial conformation and the formation of neurofibrillary tangles (NFTs). The phosphorylation status of tau at different epitopes affects its folding and propensity to aggregate (Jeganathan et al., 2008). In our study, we observed differences in the phosphorylation of the PHF1 epitope between the PI and CI female groups. The CI females display higher levels of tau phosphorylation. This suggests that chronic social isolation may create conditions that promote increased tau phosphorylation in females. Interestingly, in males, it was the re-socialized group that exhibited the highest levels of tau phosphorylation compared to the other groups. This indicates that the consequences of tau phosphorylation in males may persist even after the treatment period ends.

We also examined the phosphorylation of the AT8 epitope, which is another phosphorylation site of tau. While no differences were found among female groups, in males consistently higher levels of AT8 phosphorylation were observed in all treated males. This suggests that long-term SIS induces cellular changes that specifically impact tau folding in males. Furthermore, the observation that males showed high levels of phosphorylated PHF1 in the CI-R group indicates a greater susceptibility to tau aggregation in males. The combination of phosphorylated epitopes, including both PHF1 and AT8, is known to induce the pathological conformation of tau observed during AD development (Jeganathan et al., 2008).

Interestingly, the measurement of total Tau protein levels reveals a lower amount of total tau protein in PI females, while no changes were observed in treated males. This data suggests that there are no significant alterations in protein synthesis, but rather changes in the phosphorylation level. It indicates that social isolation primarily affects the post-translational modification of tau protein rather than its overall synthesis, similar to observed in AD.

These findings highlight the sex-specific differences in the regulation of tau phosphorylation and suggest that chronic social isolation can influence tau pathology differently in females and males. Further research is needed to understand the underlying mechanisms and the implications of these observations in the context of neurodegenerative diseases.

### 4.6. Amyloid-β formation and aggregation

According to the amyloid hypothesis, all the alterations observed after long-term SIS in both female and male degus will be explained by increased production and aggregation of Aβ_42_ peptide. The amyloid-β is a crucial component of amyloid plaques and has been associated with various diseases, including AD (Busche and Hyman, 2020). Among the two most common forms, Aβ_42_ is less abundant than Aβ_40_ and has a higher tendency to aggregate (Pauwels et al., 2012). In our study, we observed higher levels of Aβ_42_ in the chronically isolated females compared to the other groups. However, during the re-socialization period, this level became similar to the control group, suggesting that re-socialization may have a mitigating effect on the elevated levels of Aβ_42_ in females. In contrast, males exhibited an increase in Aβ_42_ during the re-socialization period, indicating a specific sensitivity in males during this time. These findings highlight the potential role of social isolation and re-socialization in modulating Aβ_42_ levels, and suggest that the effects may differ between males and females. Understanding these gender-specific differences in Aβ metabolism and accumulation can provide valuable insights into the underlying mechanisms of neurodegenerative diseases and may have implications for the development of targeted interventions.

Additionally, we used two antibodies to target specific Aβ forms. The 4G8 antibody, which targets residues 18–23 of Aβ and that also can detect Aβ aggregates (Aβ load), showed steadily and significantly increased levels of 4G8 in all groups of females. The same was observed in males. Thus, this antibody appears to be more specific elucidating differences between groups and indicating that different degrees of social isolation correlate with the amount of Aβ load present in the hippocampus of degus. Surprisingly, re-socialization was unable to reduce Aβ, suggesting that it may accumulate throughout life, possibly because re-socialization is unable to induce disaggregation of Aβ oligomers deposits.

We also used the A11 antibody, which specifically detects prefibrillar β-amyloid oligomers. Consistently, we observed higher levels of A11 in all groups of treated females compared to the control group. Similarly, in males, there was an increase in A11 levels particularly high in the CI-R group. These findings suggest that different degrees of social isolation are associated with elevated levels of prefibrillar β-amyloid oligomers. Interestingly, neither females nor males were able to effectively reduce their amyloid concentrations during re-socialization, an aspect that suggests amyloid accumulation throughout life. These observations underscore the potential long-term consequences of social isolation on amyloid accumulation, particularly in the prefibrillar β-amyloid oligomer form. The inability to effectively reduce amyloid levels during re-socialization raises important questions about the mechanisms underlying amyloid accumulation and the need for further research to better understand and address this phenomenon.

Interestingly, housing transgenic APP/PS1 mice with social mates may lead to an increased release, without a significant deposition of amyloid plaques (Liang et al., 2019). In these animals, social interaction activity promotes the clustering of astrocytes around regions where Aβ deposition occurs, delaying plaque formation. This phenomenon is associated with improved cognitive function when compared to the isolated mice and controls (Liang et al., 2019). Although these data suggest that Aβ release alone is not sufficient to induce plaque formation, it is important to note that these animals are genetically modified, resulting in significantly higher Aβ release compared to wild-type mice. Although not the same microglia protein, we measured lower levels of Iba1 in PI males and CI females. Furthermore, several species of Aβ were detected as a consequence of long-term SIS treatment, suggesting that Aβ release may induce some degree of neuronal loss in natural animals. Further studies are needed to clarify this issue.

Finally, and according to previous studies showing that Aβ and tau pathology promotes neuronal death (Yao et al., 2005; Dong et al., 2022), our preliminary data showed a reduction in the cell thickness of the CA1 region from the hippocampus in both female and male groups. Despite that, mature neurons appear not to change in females but they do it in males after chronic isolation. This also could be potentiated by the different insults caused by the inflammatory processes, oxidative stress, bioenergetics defects and accumulation of other abnormal proteins. A more exhaustive study will be required to gain a complete visualization of how neuronal viability is affected during stress paradigms.

Understanding the mechanisms underlying these changes could offer new avenues for treatment development to slow down or prevent early onset age-related cognitive decline and neurodegenerative diseases. Whether the stress induced by early-life social isolation contributes to the development of an early-aging phenotype or increases susceptibility is still an area of ongoing research (Silva-Gómez et al., 2003; Lupien et al., 2009; Pereda-Pérez et al., 2013;Shafighi et al., 2023; Xiong et al., 2023). Longitudinal studies are required to observe the long-term effects resulting from early-life causes. A report of younger degus (than the one used in our studies) isolated from postnatal day 3, agrees that anxiety and hyperactivity are common behavioral effects of mother separation and, like in our previous study, anxiety is more expressed in isolated males than females (Ukyo et al., 2023). In this context, degus serve as an exceptional animal model to study a problem that affects human beings. Social isolation induces stress and triggers a cascade of physiological effects. Moreover, in humans, stress is intertwined with other social determinants such as education, social status, and access to healthcare. When combined with physiological characteristics (e.g., comorbidities, physical status) and lifestyle factors (e.g., food intake, smoking), these elements may potentially trigger dementia and related conditions (Shafighi et al., 2023; Xiong et al., 2023). Additionally, considering the role of sex in stress-induced isolation or aging opens up new avenues of investigation, including sex-dependent gene expression, mechanisms for oxidative stress and inflammation, course of neurodegenerative diseases, and more (Hua et al., 2010; Bellanti et al., 2013; Ardekani et al., 2016; Koran et al., 2017; Caruso et al., 2018; Ruiz-Perera et al., 2018; Albert and Newhouse, 2019; Oliva et al., 2020; Bourquard et al., 2023; Martínez de Toda et al., 2023; Tranchevent et al., 2023). Future studies should incorporate these variables, utilizing a comprehensive approach to explain physiological responses.

Our study is far from being predictive of the outcomes of long-term SIS treatment. The notion that the PI animals are less affected than CI animals, or that all CI-R animals must outperform the chronically isolated ones, is far from accurate. Biology operates not-linearly. Furthermore, these effects may involve different brain regions. Social isolation during the early life stages may have significant emotional effects impacts that may remain latent until adulthood. While long-term chronic isolation shifts the physiological system into a new steady state–other than the short periods of intense stress–the cyclical pattern of isolation during childhood could potentially sensitize individuals permanently. For instance, our study indicates that LTP develops less steadily in PI males compared to other groups, but becomes significantly lower in CI-R males. In contrast, LTP in CI males resembled that of CTRL. Conversely, LTP remained unaffected in females across treatments (Rivera et al., 2020). These data suggest that the factors that alter the course of LTP in males occur early in life, but are triggered during emotional interactions (re-socialization) with peers. Additionally, males generally display poorer spatial learning performance than females (Rivera et al., 2020), suggesting that the mechanisms that subserve this type of cellular plasticity may be more vulnerable in male subjects. Conversely, early separation had a more pronounced effect on social memory in females than in males, with the most significant impact observed in PI females (Rivera et al., 2021). The consequences of isolation by the recent COVID-19 pandemic remain largely unseen, but recent reports indicate that young girls (<15 years old) experienced worse mental health outcomes compared to young boys (Mendolia et al., 2022). Therefore, the cellular mechanisms underlying spatial learning performance and social/emotional memory are differentially affected by long-term social isolation, and the effects are sex-dependent. The effects of SIS during childhood have also been observed in other studies using different animal models (Weiss et al., 2004; Ferdman et al., 2007; Fone and Porkess, 2008; Mumtaz et al., 2018).

There is also no reason to expect that CI-R would lead to recovery at the behavioral, cellular, or molecular level. Strikingly, oxytocin and its downstream signaling remain persistently altered in multiple brain regions across all long-term SIS treatments, even though the animals exhibit recovery from social memory impairment (Rivera et al., 2021). Our data indicated that behavioral recovery during re-socialization is more common than recovery at the cellular or molecular level. This observation suggests that biological systems employ homeostatic mechanisms to overcome deficits, and would be true at least during the time period of our study. These findings underscore the need to consider multiple factors when evaluating different variables and drawing conclusions.

Altogether this study allows us to conclude that social isolation is a critical stress factor that affects severely female and male degus, activating different neurotoxic and neurodegenerative events such as inflammation, ATP deficits, ROS overproduction, and oxidative damage. In addition, considering that degus are a natural model of AD, long-term SIS also promotes an accelerated apparition of AD-related markers, such as phosphorylated tau and amyloid production and aggregation. By understanding the contribution of environmental stressors experienced early in life to the susceptibility to the later development of Alzheimer’s neuropathology, this study may pave the way to more precise treatments and even strategies to halt or delay the “spread” of this debilitating disease.

## Data availability statement

The raw data supporting the conclusions of this article are available by the corresponding authors upon reasonable request.

## Ethics statement

All animal protocols followed the National Institutes of Health guide for the care and use of laboratory animals (NIH Publications No. 8023, revised 1978). All procedures were approved by the Bioethical and Biosafety Committee of the Faculty of Biological Sciences of the Pontificia Universidad Católica de Chile (CBB-121-2013). Efforts were made to minimize animal suffering and to reduce the number of animals used. The study was conducted in accordance with the local legislation and institutional requirements.

## Author contributions

CAO, CT-R, and DSR: conceptualization, methodology, and validation. CAO, ML, CJ, AC, CT-R, and DSR: formal analysis. CAO, ML, CJ, AC, CBL, CT-R, and DSR: investigation. CAO and DSR: writing—original draft preparation. CAO, TAM, CBL, GC, NCI, CT-R, and DSR: writing—review and editing. DSR, TAM, FB, NCI, and CT-R: funding acquisition. All authors have read and agreed to the published version of the manuscript.

## Funding

This work was supported by a grant from Fondo Nacional de Desarrollo Científico y Tecnológico (FONDECYT) No. 11190603 to DR, No. 11220157 to TAM, and No. 1221178 to CT-R. In addition, a grant from ANID PIA/BASAL FB0002 was awarded to FB and GC and Centro Ciencia & Vida, FB210008, Financiamiento Basal para Centros Científicos y Tecnológicos de Excelencia de ANID to CT-R. We thank the helpful collaboration of the FdI Grant UAU22101 from the Ministerio de Educación, Gobierno de Chile, to the Universidad Autónoma de Chile.

## Conflict of interest

The authors declare that the research was conducted in the absence of any commercial or financial relationships that could be construed as a potential conflict of interest.

## Publisher’s note

All claims expressed in this article are solely those of the authors and do not necessarily represent those of their affiliated organizations, or those of the publisher, the editors and the reviewers. Any product that may be evaluated in this article, or claim that may be made by its manufacturer, is not guaranteed or endorsed by the publisher.

## References

[ref2] Aigelsreiter A. Janig E. Stumptner C. Fuchsbichler A. Zatloukal K. Denk H. (2007). How a cell deals with abnormal proteins. Pathogenetic mechanisms in protein aggregation diseases. Pathobiology 74, 145–158. doi: 10.1159/000103374 17643060

[ref3] Albert K. M. Newhouse P. A. (2019). Estrogen, stress, and depression: cognitive and biological interactions. Annu. Rev. Clin. Psychol. 15, 399–423. doi: 10.1146/annurev-clinpsy-050718-095557, PMID: 30786242 PMC9673602

[ref4] Ardekani B. A. Convit A. Bachman A. H. (2016). Analysis of the MIRIAD data shows sex differences in hippocampal atrophy progression. J. Alzheimers Dis. 50, 847–857. doi: 10.3233/JAD-150780, PMID: 26836168

[ref5] Armstrong R. (2019). Risk factors for Alzheimer’s disease. Folia Neuropathol. 57, 87–105. doi: 10.5114/fn.2019.85929 31556570

[ref6] Bellanti F. Matteo M. Rollo T. De Rosario F. Greco P. Vendemiale G.. (2013). Sex hormones modulate circulating antioxidant enzymes: impact of estrogen therapy. Redox Biol. 1, 340–346. doi: 10.1016/j.redox.2013.05.003, PMID: 24024169 PMC3757703

[ref7] Bernardi S. Toffoli B. Tonon F. Francica M. Campagnolo E. Ferretti T.. (2020). Sex differences in Proatherogenic cytokine levels. Int. J. Mol. Sci. 21. doi: 10.3390/ijms21113861, PMID: 32485823 PMC7311959

[ref8] Bourquard T. Lee K. al-Ramahi I. Pham M. Shapiro D. Lagisetty Y.. (2023). Functional variants identify sex-specific genes and pathways in Alzheimer’s disease. Nat. Commun. 14, 2765–2715. doi: 10.1038/s41467-023-38374-z, PMID: 37179358 PMC10183026

[ref9] Breitzig M. Bhimineni C. Lockey R. Kolliputi N. (2016). 4-Hydroxy-2-nonenal: a critical target in oxidative stress? Am. J. Physiol. Cell Physiol. 311, C537–C543. doi: 10.1152/ajpcell.00101.2016, PMID: 27385721 PMC5129750

[ref10] Bronzuoli M. R. Iacomino A. Steardo L. Scuderi C. (2016). Targeting neuroinflammation in Alzheimer’s disease. J. Inflamm. Res. 9, 199–208. doi: 10.2147/JIR.S86958, PMID: 27843334 PMC5098782

[ref11] Busche M. A. Hyman B. T. (2020). Synergy between amyloid-β and tau in Alzheimer’s disease. Nat. Neurosci. 23, 1183–1193. doi: 10.1038/s41593-020-0687-6 32778792 PMC11831977

[ref12] Carroll J. C. Iba M. Bangasser D. A. Valentino R. J. James M. J. Brunden K. R.. (2011). Chronic stress exacerbates tau pathology, neurodegeneration, and cognitive performance through a Corticotropin-releasing factor receptor-dependent mechanism in a transgenic mouse model of Tauopathy. J. Neurosci. 31, 14436–14449. doi: 10.1523/JNEUROSCI.3836-11.2011, PMID: 21976528 PMC3230070

[ref13] Caruso A. Nicoletti F. Mango D. Saidi A. Orlando R. Scaccianoce S. (2018). Stress as risk factor for Alzheimer’s disease. Pharmacol. Res. 132, 130–134. doi: 10.1016/j.phrs.2018.04.017 29689315

[ref14] Chunhui H. Dilin X. Ke Z. Jieyi S. Sicheng Y. Dapeng W.. (2018). A11-positive β-amyloid oligomer preparation and assessment using dot blotting analysis. J. Vis. Exp. 2018 10.3791/57592 PMC6101355 29889206

[ref15] Coppedè F. Mancuso M. Siciliano G. Migliore L. Murri L. (2006). Genes and the environment in neurodegeneration. Biosci. Rep. 26, 341–367. doi: 10.1007/s10540-006-9028-6 17029001

[ref16] Curto M. Martocchia A. Ferracuti S. Comite F. Scaccianoce S. Girardi P.. (2017). Increased Total urinary cortisol (tUC) and serum brain-derived neurotrophic factor (BDNF) ratio in Alzheimer disease (AD)-affected patients. Alzheimer Dis. Assoc. Disord. 31, 173–176. doi: 10.1097/WAD.0000000000000156, PMID: 27196536

[ref17] Deponte M. (2013). Glutathione catalysis and the reaction mechanisms of glutathione-dependent enzymes. Biochim. Biophys. Acta 1830, 3217–3266. doi: 10.1016/j.bbagen.2012.09.018, PMID: 23036594

[ref18] Dinarello C. A. Simon A. Van Der Meer J. W. M. (2012). Treating inflammation by blocking interleukin-1 in a broad spectrum of diseases. Nat. Rev. Drug Discov. 11, 633–652. doi: 10.1038/nrd3800, PMID: 22850787 PMC3644509

[ref19] Dong Y. Yu H. Li X. Bian K. Zheng Y. Dai M.. (2022). Hyperphosphorylated tau mediates neuronal death by inducing necroptosis and inflammation in Alzheimer’s disease. J. Neuroinflammation 19:205. doi: 10.1186/s12974-022-02567-y, PMID: 35971179 PMC9377071

[ref20] Dröge W. Schipper H. M. (2007). Oxidative stress and aberrant signaling in aging and cognitive decline. Aging Cell 6, 361–370. doi: 10.1111/j.1474-9726.2007.00294.x, PMID: 17517043 PMC1974775

[ref21] Dromard Y. Arango-Lievano M. Borie A. Dedin M. Fontanaud P. Torrent J.. (2022). Loss of glucocorticoid receptor phosphorylation contributes to cognitive and neurocentric damages of the amyloid-β pathway. Acta Neuropathol. Commun. 10:91. doi: 10.1186/s40478-022-01396-7, PMID: 35733193 PMC9219215

[ref22] Dunn A. R. O’Connell K. M. S. Kaczorowski C. C. (2019). Gene-by-environment interactions in Alzheimer’s disease and Parkinson’s disease. Neurosci. Biobehav. Rev. 103, 73–80. doi: 10.1016/j.neubiorev.2019.06.018, PMID: 31207254 PMC6700747

[ref23] Dwivedi D. Megha K. Mishra R. Mandal P. K. (2020). Glutathione in brain: overview of its conformations, functions, biochemical characteristics, quantitation and potential therapeutic role in brain disorders. Neurochem. Res. 45, 1461–1480. doi: 10.1007/s11064-020-03030-1, PMID: 32297027

[ref24] Ferdman N. Murmu R. P. Bock J. Braun K. Leshem M. (2007). Weaning age, social isolation, and gender, interact to determine adult explorative and social behavior, and dendritic and spine morphology in prefrontal cortex of rats. Behav. Brain Res. 180, 174–182. doi: 10.1016/j.bbr.2007.03.011, PMID: 17477981

[ref25] Fone K. C. F. Porkess M. V. (2008). Behavioural and neurochemical effects of post-weaning social isolation in rodents-relevance to developmental neuropsychiatric disorders. Neurosci. Biobehav. Rev. 32, 1087–1102. doi: 10.1016/j.neubiorev.2008.03.003, PMID: 18423591

[ref26] Futch H. S. Croft C. L. Truong V. Q. Krause E. G. Golde T. E. (2017). Targeting psychologic stress signaling pathways in Alzheimer’s disease. Mol. Neurodegener. 12:49. doi: 10.1186/s13024-017-0190-z, PMID: 28633663 PMC5479037

[ref27] Green K. N. Billings L. M. Roozendaal B. McGaugh J. L. LaFerla F. M. (2006). Glucocorticoids increase amyloid-β and tau pathology in a mouse model of Alzheimer’s disease. J. Neurosci. 26, 9047–9056. doi: 10.1523/JNEUROSCI.2797-06.2006, PMID: 16943563 PMC6675335

[ref28] Gubert C. Kong G. Renoir T. Hannan A. J. (2020). Exercise, diet and stress as modulators of gut microbiota: implications for neurodegenerative diseases. Neurobiol. Dis. 134:104621. doi: 10.1016/j.nbd.2019.104621, PMID: 31628992

[ref29] Hatami A. Monjazeb S. Glabe C. (2016). The anti-amyloid-β monoclonal antibody 4G8 recognizes a generic sequence-independent epitope associated with α-Synuclein and islet amyloid polypeptide amyloid fibrils. J. Alzheimers Dis. 50, 517–525. doi: 10.3233/JAD-150696, PMID: 26682688

[ref30] Hua X. Hibar D. P. Lee S. Toga A. W. Jack C. R. Weiner M. W.. (2010). Sex and age differences in atrophic rates: an ADNI study with n=1368 MRI scans. Neurobiol. Aging 31, 1463–1480. doi: 10.1016/j.neurobiolaging.2010.04.033, PMID: 20620666 PMC2927200

[ref31] Hurley M. J. Deacon R. M. J. Beyer K. Ioannou E. Ibáñez A. Teeling J. L.. (2018). The long-lived *Octodon degus* as a rodent drug discovery model for Alzheimer’s and other age-related diseases. Pharmacol. Ther. 188, 36–44. doi: 10.1016/j.pharmthera.2018.03.001, PMID: 29514054

[ref32] Inestrosa N. C. Reyes A. E. Chacón M. A. Cerpa W. Villalón A. Montiel J.. (2005). Human-like rodent amyloid-β-peptide determines Alzheimer pathology in aged wild-type Octodon degu. Neurobiol. Aging 26, 1023–1028. doi: 10.1016/j.neurobiolaging.2004.09.016 15748782

[ref33] Jeganathan S. Hascher A. Chinnathambi S. Biernat J. Mandelkow E.-M. Mandelkow E. (2008). Proline-directed pseudo-phosphorylation at AT8 and PHF1 epitopes induces a compaction of the paperclip folding of tau and generates a pathological (MC-1) conformation. J. Biol. Chem. 283, 32066–32076. doi: 10.1074/jbc.M805300200, PMID: 18725412

[ref34] Jha N. K. Jha S. K. Kar R. Nand P. Swati K. Goswami V. K. (2019). Nuclear factor-kappa β as a therapeutic target for Alzheimer’s disease. J. Neurochem. 150, 113–137. doi: 10.1111/jnc.14687 30802950

[ref35] Karl J. P. Hatch A. M. Arcidiacono S. M. Pearce S. C. Pantoja-Feliciano I. G. Doherty L. A.. (2018). Effects of psychological, environmental and physical stressors on the Gut Microbiota. Front. Microbiol. 9:13. doi: 10.3389/fmicb.2018.02013 30258412 PMC6143810

[ref36] Kline S. A. Mega M. S. (2020). Stress-induced neurodegeneration: the potential for coping as neuroprotective therapy. Am. J. Alzheimers Dis. Other Dement. 35:153331752096087. doi: 10.1177/1533317520960873 PMC10623922 32969239

[ref37] Koran M. E. I. Wagener M. Hohman T. J. (2017). Sex differences in the association between AD biomarkers and cognitive decline. Brain Imaging Behav. 11, 205–213. doi: 10.1007/s11682-016-9523-8, PMID: 26843008 PMC4972701

[ref38] Kregel K. C. Zhang H. J. (2007). An integrated view of oxidative stress in aging: basic mechanisms, functional effects, and pathological considerations. Am. J. Physiol. Regul. Integr. Comp. Physiol. 292, R18–R36. doi: 10.1152/ajpregu.00327.2006, PMID: 16917020

[ref39] Laforge M. Rodrigues V. Silvestre R. Gautier C. Weil R. Corti O.. (2016). NF-κB pathway controls mitochondrial dynamics. Cell Death Differ. 23, 89–98. doi: 10.1038/cdd.2015.42, PMID: 26024391 PMC4815975

[ref40] Leal M. C. Casabona J. C. Puntel M. Pitossi F. (2013). Interleukin-1beta and TNF-alpha: reliable targets for protective therapies in Parkinson’s disease? Front. Cell. Neurosci. 7, 1–10. doi: 10.3389/fncel.2013.00053 23641196 PMC3638129

[ref41] Lennicke C. Cochemé H. M. (2021). Redox metabolism: ROS as specific molecular regulators of cell signaling and function. Mol. Cell 81, 3691–3707. doi: 10.1016/j.molcel.2021.08.018, PMID: 34547234

[ref42] Levy D. R. Yizhar O. (2018). Stress and sociability. Nat. Neurosci. 21, 304–306. doi: 10.1038/s41593-018-0088-2, PMID: 29476129

[ref43] Liang F. Yang S. Zhang Y. Hao T. (2019). Social housing promotes cognitive function through enhancing synaptic plasticity in APP/PS1 mice. Behav. Brain Res. 368:111910. doi: 10.1016/j.bbr.2019.111910, PMID: 31034995

[ref44] Lindsay C. B. Zolezzi J. M. Rivera D. S. Cisternas P. Bozinovic F. Inestrosa N. C. (2020). Andrographolide reduces Neuroinflammation and oxidative stress in aged *Octodon degus*. Mol. Neurobiol. 57, 1131–1145. doi: 10.1007/s12035-019-01784-6, PMID: 31701436

[ref45] Lingappan K. (2018). NF-κB in oxidative stress. Curr Opin Toxicol 7, 81–86. doi: 10.1016/j.cotox.2017.11.002, PMID: 29862377 PMC5978768

[ref46] Lupien S. J. McEwen B. S. Gunnar M. R. Heim C. (2009). Effects of stress throughout the lifespan on the brain, behaviour and cognition. Nat. Rev. Neurosci. 10, 434–445. doi: 10.1038/nrn2639 19401723

[ref47] Martínez de Toda I. González-Sánchez M. Díaz-Del Cerro E. Valera G. Carracedo J. Guerra-Pérez N. (2023). Sex differences in markers of oxidation and inflammation implications for ageing. Mech. Ageing Dev. 211:111797. doi: 10.1016/j.mad.2023.111797 36868323

[ref1001] Matrisciano F. Pinna G. (2021). PPAR-α Hypermethylation in the Hippocampus of Mice Exposed to Social Isolation Stress Is Associated with Enhanced Neuroinflammation and Aggressive Behavior. Int. J. Mol. Sci. 22:10678. 34639019 10.3390/ijms221910678 PMC8509148

[ref48] Mendolia S. Suziedelyte A. Zhu A. (2022). Have girls been left behind during the COVID-19 pandemic? Gender differences in pandemic effects on children’s mental wellbeing. Econ. Lett. 214:110458. doi: 10.1016/j.econlet.2022.110458, PMID: 35345669 PMC8944107

[ref49] Möller M. Du Preez J. L. Viljoen F. P. Berk M. Emsley R. Harvey B. H. (2013). Social isolation rearing induces mitochondrial, immunological, neurochemical and behavioural deficits in rats, and is reversed by clozapine or N-acetyl cysteine. Brain Behav. Immun. 30, 156–167. doi: 10.1016/j.bbi.2012.12.011 23270677

[ref50] Mumtaz F. Khan M. I. Zubair M. Dehpour A. R. (2018). Neurobiology and consequences of social isolation stress in animal model—a comprehensive review. Biomed. Pharmacother. 105, 1205–1222. doi: 10.1016/j.biopha.2018.05.086, PMID: 30021357

[ref51] Muntsant A. Giménez-Llort L. (2022). Crosstalk of Alzheimer’s disease-phenotype, HPA axis, splenic oxidative stress and frailty in late-stages of dementia, with special concerns on the effects of social isolation: a translational neuroscience approach. Front. Aging Neurosci. 14, 1–13. doi: 10.3389/fnagi.2022.969381 PMC9520301 36185472

[ref52] Murphy M. P. Bayir H. Belousov V. Chang C. J. Davies K. J. A. Davies M. J.. (2022). Guidelines for measuring reactive oxygen species and oxidative damage in cells and in vivo. Nat. Metab. 4, 651–662. doi: 10.1038/s42255-022-00591-z, PMID: 35760871 PMC9711940

[ref53] Nandi A. Yan L.-J. Jana C. K. Das N. (2019). Role of catalase in oxidative stress- and age-associated degenerative diseases. Oxidative Med. Cell. Longev. 2019, 1–19. doi: 10.1155/2019/9613090 PMC6885225 31827713

[ref54] Niveditha S. Deepashree S. Ramesh S. R. Shivanandappa T. (2017). Sex differences in oxidative stress resistance in relation to longevity in *Drosophila melanogaster*. J. Comp. Physiol. B 187, 899–909. doi: 10.1007/s00360-017-1061-1, PMID: 28261744

[ref55] Olesen M. A. Torres A. K. Jara C. Murphy M. P. Tapia-Rojas C. (2020). Premature synaptic mitochondrial dysfunction in the hippocampus during aging contributes to memory loss. Redox Biol. 34:101558. doi: 10.1016/j.redox.2020.101558, PMID: 32447261 PMC7248293

[ref1002] Oliva M. Muñoz-Aguirre M. Kim-Hellmuth S. Wucher V. Gewirtz A. D. H. Cotter D. J.. (2020). The impact of sex on gene expression across human tissues. Science 369, eaba3066. doi: 10.1126/science.aba3066 32913072 PMC8136152

[ref56] Ouanes S. Popp J. (2019). High cortisol and the risk of dementia and Alzheimer’s disease: a review of the literature. Front. Aging Neurosci. 11:43. doi: 10.3389/fnagi.2019.00043, PMID: 30881301 PMC6405479

[ref57] Park M. H. Hong J. T. (2016). Roles of NF-κB in Cancer and inflammatory diseases and their therapeutic approaches. Cells 5:15. doi: 10.3390/cells5020015, PMID: 27043634 PMC4931664

[ref58] Pauwels K. Williams T. L. Morris K. L. Jonckheere W. Vandersteen A. Kelly G.. (2012). Structural basis for increased toxicity of pathological aβ42:aβ40 ratios in Alzheimer disease. J. Biol. Chem. 287, 5650–5660. doi: 10.1074/jbc.M111.264473 22157754 PMC3285338

[ref59] Pereda-Pérez I. Popović N. Otalora B. B. Popović M. Madrid J. A. Rol M. A.. (2013). Long-term social isolation in the adulthood results in CA1 shrinkage and cognitive impairment. Neurobiol. Learn. Mem. 106, 31–39. doi: 10.1016/j.nlm.2013.07.004, PMID: 23867635

[ref60] Peskind E. R. Wilkinson C. W. Petrie E. C. Schellenberg G. D. Raskind M. A. (2001). Increased CSF cortisol in AD is a function of APOE genotype. Neurology 56, 1094–1098. doi: 10.1212/WNL.56.8.1094, PMID: 11320185

[ref61] Prince M. Acosta D. Ferri C. P. Guerra M. Huang Y. Rodriguez J. J. L.. (2012). Dementia incidence and mortality in middle-income countries, and associations with indicators of cognitive reserve: a 10/66 dementia research group population-based cohort study. Lancet (London, England) 380, 50–58. doi: 10.1016/S0140-6736(12)60399-7, PMID: 22626851 PMC3525981

[ref62] Ricci S. Fuso A. Ippoliti F. Businaro R. (2012). Stress-induced cytokines and neuronal dysfunction in Alzheimer’s disease. J. Alzheimers Dis. 28, 11–24. doi: 10.3233/JAD-2011-110821, PMID: 22124029

[ref63] Rivera D. S. Lindsay C. B. Oliva C. A. Bozinovic F. Inestrosa N. C. (2021). “Live together, die alone”: the effect of re-socialization on behavioural performance and social-affective brain-related proteins after a long-term chronic social isolation stress. Neurobiol. Stress 14:100289. doi: 10.1016/j.ynstr.2020.100289, PMID: 33426200 PMC7785960

[ref64] Rivera D. S. Lindsay C. B. Oliva C. A. Codocedo J. F. Bozinovic F. Inestrosa N. C. (2020). Effects of long-lasting social isolation and re-socialization on cognitive performance and brain activity: a longitudinal study in *Octodon degus*. Sci. Rep. 10:18315. doi: 10.1038/s41598-020-75026-4, PMID: 33110163 PMC7591540

[ref65] Ross C. A. Poirier M. A. (2004). Protein aggregation and neurodegenerative disease. Nat. Med. 10, S10–S17. doi: 10.1038/nm1066 15272267

[ref66] Ruiz-Perera L. M. Schneider L. Windmöller B. A. Müller J. Greiner J. F. W. Kaltschmidt C.. (2018). NF-κB p65 directs sex-specific neuroprotection in human neurons. Sci. Rep. 8:16012. doi: 10.1038/s41598-018-34394-8, PMID: 30375448 PMC6207661

[ref67] Sasaki Y. Ohsawa K. Kanazawa H. Kohsaka S. Imai Y. (2001). Iba1 is an actin-cross-linking protein in macrophages/microglia. Biochem. Biophys. Res. Commun. 286, 292–297. doi: 10.1006/bbrc.2001.5388, PMID: 11500035

[ref68] Scheiblich H. Trombly M. Ramirez A. Heneka M. T. (2020). Neuroimmune connections in aging and neurodegenerative diseases. Trends Immunol. 41, 300–312. doi: 10.1016/j.it.2020.02.002, PMID: 32147113

[ref69] Scheltens P. Blennow K. Breteler M. M. B. de Strooper B. Frisoni G. B. Salloway S.. (2016). Alzheimer’s disease. Lancet 388, 505–517. doi: 10.1016/S0140-6736(15)01124-1 26921134

[ref70] Schieber M. Chandel N. S. (2014). ROS function in redox signaling and oxidative stress. Curr. Biol. 24, R453–R462. doi: 10.1016/j.cub.2014.03.034, PMID: 24845678 PMC4055301

[ref71] Selkoe D. J. Hardy J. (2016). The amyloid hypothesis of Alzheimer’s disease at 25 years. EMBO Mol. Med. 8, 595–608. doi: 10.15252/emmm.201606210, PMID: 27025652 PMC4888851

[ref72] Shafighi K. Villeneuve S. Rosa Neto P. Badhwar A. P. Poirier J. Sharma V.. (2023). Social isolation is linked to classical risk factors of Alzheimer’s disease-related dementias. PLoS One 18, e0280471–e0280420. doi: 10.1371/journal.pone.0280471, PMID: 36724157 PMC9891507

[ref73] Shapiro L. A. Perez Z. D. Foresti M. L. Arisi G. M. Ribak C. E. (2009). Morphological and ultrastructural features of Iba1-immunolabeled microglial cells in the hippocampal dentate gyrus. Brain Res. 1266, 29–36. doi: 10.1016/j.brainres.2009.02.031 19249294 PMC2677570

[ref74] Shir D. Graff-Radford J. Hofrenning E. I. Lesnick T. G. Przybelski S. A. Lowe V. J.. (2022). Association of plasma glial fibrillary acidic protein (GFAP) with neuroimaging of Alzheimer’s disease and vascular pathology. Alzheimers Dement. 14:e12291. doi: 10.1002/dad2.12291, PMID: 35252538 PMC8883441

[ref75] Shoeb M. Ansari N. Srivastava S. Ramana K. (2013). 4-Hydroxynonenal in the pathogenesis and progression of human diseases. Curr. Med. Chem. 21, 230–237. doi: 10.2174/09298673113209990181, PMID: 23848536 PMC3964795

[ref76] Silva-Gómez A. B. Rojas D. Juárez I. Flores G. (2003). Decreased dendritic spine density on prefrontal cortical and hippocampal pyramidal neurons in postweaning social isolation rats. Brain Res. 983, 128–136. doi: 10.1016/S0006-8993(03)03042-7, PMID: 12914973

[ref77] Spires-Jones T. L. Attems J. Thal D. R. (2017). Interactions of pathological proteins in neurodegenerative diseases. Acta Neuropathol. 134, 187–205. doi: 10.1007/s00401-017-1709-7, PMID: 28401333 PMC5508034

[ref78] Tapia-Rojas C. Cabezas-Opazo F. Deaton C. A. Vergara E. H. Johnson G. V. W. Quintanilla R. A. (2019). It’s all about tau. Prog. Neurobiol. 175, 54–76. doi: 10.1016/j.pneurobio.2018.12.005, PMID: 30605723 PMC6397676

[ref79] Tapia-Rojas C. Inestrosa N. C. (2018). Wnt signaling loss accelerates the appearance of neuropathological hallmarks of Alzheimer’s disease in J20-APP transgenic and wild-type mice. J. Neurochem. 144, 443–465. doi: 10.1111/jnc.14278, PMID: 29240990

[ref80] Tarragon E. Lopez D. Estrada C. Ana G.-C. Schenker E. Pifferi F.. (2013). *Octodon degus*: a model for the cognitive impairment associated with Alzheimer’s disease. CNS Neurosci. Ther. 19, 643–648. doi: 10.1111/cns.12125, PMID: 23710760 PMC6493546

[ref81] Torres A. K. Jara C. Llanquinao J. Lira M. Cortés-Díaz D. Tapia-Rojas C. (2023). Mitochondrial bioenergetics, redox balance, and calcium homeostasis dysfunction with defective ultrastructure and quality control in the Hippocampus of aged female C57BL/6J mice. Int. J. Mol. Sci. 24:5476. doi: 10.3390/ijms24065476 36982549 PMC10056753

[ref82] Torres A. K. Jara C. Olesen M. A. Tapia-Rojas C. (2021). Pathologically phosphorylated tau at S396/404 (PHF-1) is accumulated inside of hippocampal synaptic mitochondria of aged wild-type mice. Sci. Rep. 11:4448. doi: 10.1038/s41598-021-83910-w, PMID: 33627790 PMC7904815

[ref83] Tranchevent L.-C. Halder R. Glaab E. (2023). Systems level analysis of sex-dependent gene expression changes in Parkinson’s disease. Npj Park. Dis. 9:8. doi: 10.1038/s41531-023-00446-8, PMID: 36681675 PMC9867746

[ref84] Ukyo R. Shinohara A. Koshimoto C. Nagura-Kato G. A. Ieiri S. Tsuzuki Y.. (2023). Long-term behavioral effects of social separation during early life in a social mammal Octodon degus. Sci. Rep. 13:9518. doi: 10.1038/s41598-023-36745-6, PMID: 37308511 PMC10261089

[ref85] van Groen T. Kadish I. Popović N. Popović M. Caballero-Bleda M. Baño-Otálora B.. (2011). Age-related brain pathology in Octodon degu: blood vessel, white matter and Alzheimer-like pathology. Neurobiol. Aging 32, 1651–1661. doi: 10.1016/j.neurobiolaging.2009.10.008, PMID: 19910078

[ref86] Wang H. Yang F. Zhang S. Xin R. Sun Y. (2021). Genetic and environmental factors in Alzheimer’s and Parkinson’s diseases and promising therapeutic intervention via fecal microbiota transplantation. Npj Park. Dis. 7:70. doi: 10.1038/s41531-021-00213-7, PMID: 34381040 PMC8357954

[ref87] Weiss I. C. Pryce C. R. Jongen-Rêlo A. L. Nanz-Bahr N. I. Feldon J. (2004). Effect of social isolation on stress-related behavioural and neuroendocrine state in the rat. Behav. Brain Res. 152, 279–295. doi: 10.1016/j.bbr.2003.10.015 15196796

[ref88] Xiong Y. Hong H. Liu C. Zhang Y. Q. (2023). Social isolation and the brain: effects and mechanisms. Mol. Psychiatry 28, 191–201. doi: 10.1038/s41380-022-01835-w, PMID: 36434053 PMC9702717

[ref89] Yao M. Nguyen T.-V. V. Pike C. J. (2005). β-Amyloid-induced neuronal apoptosis involves c-Jun N-terminal kinase-dependent downregulation of Bcl-w. J. Neurosci. 25, 1149–1158. doi: 10.1523/JNEUROSCI.4736-04.2005, PMID: 15689551 PMC6725978

