# Supplementary material for: Long-term social isolation stress exacerbates sex-specific neurodegeneration markers in a natural model of Alzheimer’s disease
Source: Front Aging Neurosci. 2023 Sep 20;15:1250342. doi: 10.3389/fnagi.2023.1250342 (PMC10557460; doi:10.3389/fnagi.2023.1250342)
Supplement: Supplementary file 1 [file Image_1.pdf]

Supplementary Material

Figure 1S

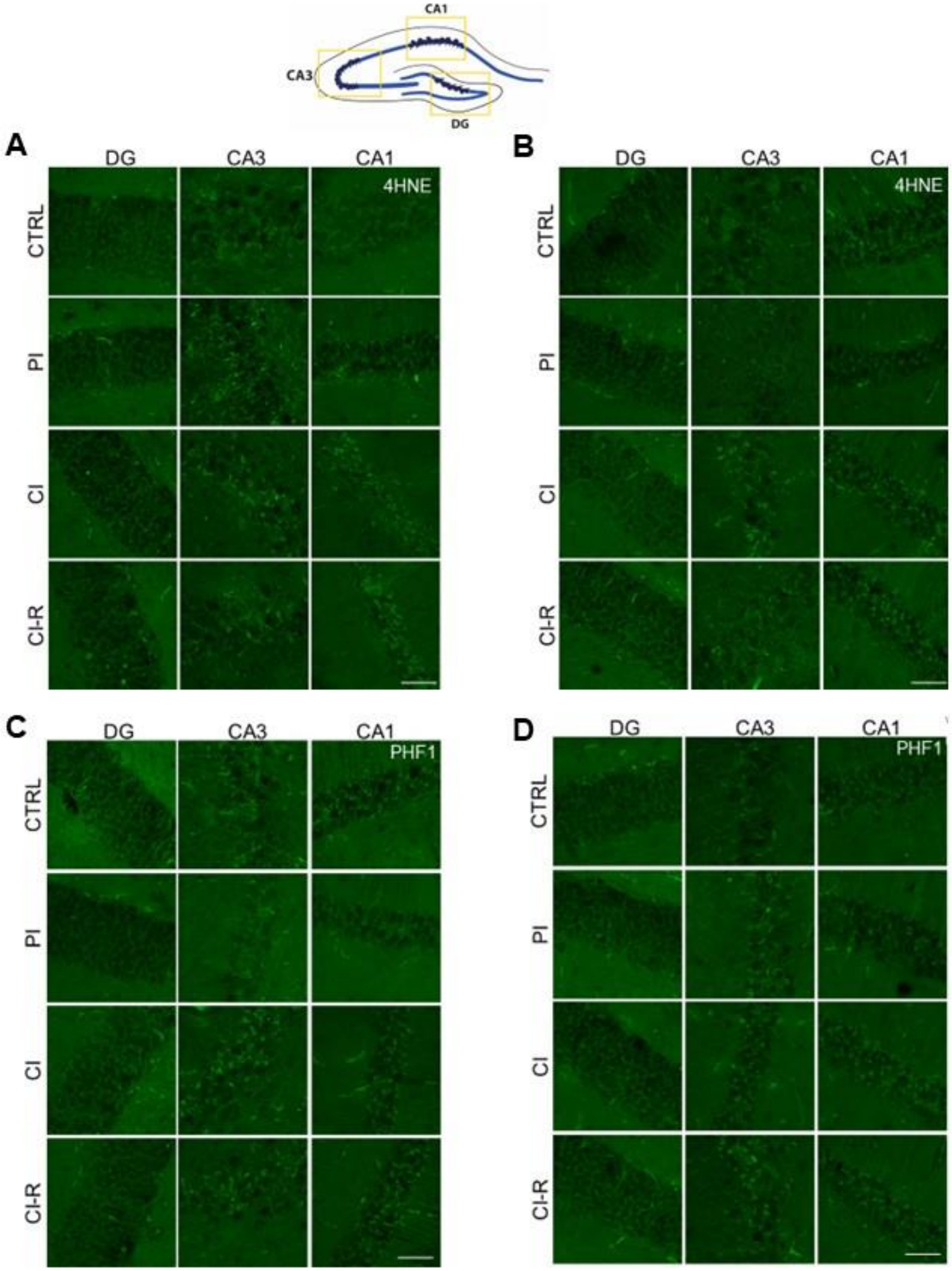

Figure Legend

Figure 1S. Immunofluorescence for oxidative damage and phosphorylated tau in PHF-1 epitope (Ser396 and Ser404) in the hippocampus of female and male degus. (A,B) Indirect immunofluorescence using the 4HNE antibody to evaluate the oxidative damage presented in the Dentate Gyrus (DG), CA1, and CA3 regions from the hippocampus of female (A) and male (B) degus.(C,D) Indirect immunofluorescence using the anti-tau PHF1 antibody (Ser396 and Ser404) to evaluate the tau phosphorylation presented in the DG, CA1, and CA3 regions from the hippocampus of female (C) and male (D) degus. Images were obtained using confocal microscopy. Representative images at 63x of one female and male at every condition. Bar scale = 50 μm.
